# *Etv5* Is Required for Peripheral Nerve Function and the Injury Response

**DOI:** 10.1523/ENEURO.0410-20.2025

**Published:** 2025-07-10

**Authors:** Lauren Belfiore, Anjali Balakrishnan, Yohannes Soenjaya, Hussein Ghazale, Alexandra Moffat, Dawn Zinyk, Lakshmy Vasan, Yacine Touahri, Yutaka Amemiya, Tak-Ho Chu, Morgan G. Stykel, Arun Seth, Satoshi Okawa, Christine E. M. Demore, Rajiv Midha, Jeff Biernaskie, Carol Schuurmans

**Affiliations:** ^1^Sunnybrook Research Institute, Toronto, Ontario M4N 3M5, Canada; ^2^Department of Laboratory Medicine and Pathobiology, University of Toronto, Toronto, Ontario M5S 3K3, Canada; ^3^Department of Biochemistry, University of Toronto, Toronto, Ontario M5S 1A8, Canada; ^4^Department of Biochemistry and Molecular Biology, University of Calgary, Calgary, Alberta T2N 1N4, Canada; ^5^Department of Ophthalmology and Vision Sciences, University of Toronto, Toronto, Ontario M5T 3A9, Canada; ^6^Department of Clinical Neurosciences, University of Calgary, Calgary, Alberta T2N 4N1, Canada; ^7^Hotchkiss Brain Institute, University of Calgary, Calgary, Alberta T2N 4N1, Canada; ^8^Department of Comparative Biology and Experimental Medicine, University of Calgary, Calgary, Alberta T2N 1N4, Canada; ^9^Pittsburgh Heart, Lung, and Blood Vascular Medicine Institute, University of Pittsburgh School of Medicine, Pittsburgh, Pennsylvania 15213; ^10^Department of Computational and Systems Biology, University of Pittsburgh School of Medicine, Pittsburgh, Pennsylvania 15260; ^11^McGowan Institute for Regenerative Medicine, University of Pittsburgh School of Medicine, Pittsburgh, Pennsylvania 15219; ^12^Department of Medical Biophysics, University of Toronto, Toronto, Ontario M5G 2C4, Canada

**Keywords:** development, *Etv5*, peripheral nerve injury, Schwann cell maturation, Schwann cell repair, Schwann cells

## Abstract

The development of Schwann cells, which myelinate axons in the peripheral nervous system, is critically dependent on MEK/ERK signaling. While Ets-domain transcription factors (*Etv1*, *Etv4*, *Etv5*) are downstream effectors of this pathway, only *Etv1* has been specifically linked to Schwann cell development. Here, we examined the functions of *Etv5*, which is expressed in Schwann cell precursors, neural crest cells and satellite glia, at embryonic stages and at low levels in mature Schwann cells. In hypomorphic *Etv5^tm1Kmm^* homozygous mutant mice, no overt defects in Schwann cell differentiation were observed at embryonic stages. To study the function of *Etv5* in juvenile (postnatal days 21–30) and mature adult (6 month) mice, we generated *Etv5* conditional knock-outs (cKOs) using a *Sox10-Cre* driver. In juvenile male *Etv5*-cKO mice, Schwann cell numbers increased normally after a peripheral nerve crush injury, a response that was attenuated by 6 months. Transmission electron microscopy of the naive sciatic nerve revealed a decline in axonal diameter and perturbed myelination in *Etv5*-cKO male and female mice. The innervated gastrocnemius muscle declined in area and volume in *Etv5*-cKO mice of both sexes, suggesting nerve structural abnormalities cause muscle atrophy. However, control and *Etv5*-cKO male and female mice performed similarly in motor behavior tests after a crush injury. Thus, *Etv5* is not essential for Schwann cell differentiation, but *Etv5* plays a crucial role in the age-dependent regulation of Schwann cell function, including nerve repair and the maintenance of axonal integrity in mature peripheral nerves.

## Graphical abstract

**Figure:**
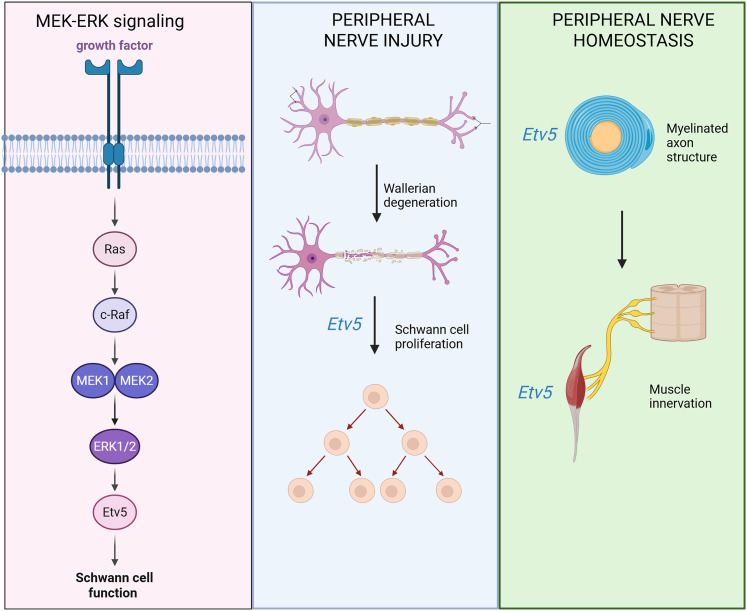
Visual Abstract

## Significance Statement

This study demonstrates that *Etv5* is crucial for Schwann cell development, myelination, and nerve health. Using *Etv5*-conditional knock-out (cKO) mice, we show that the loss of *Etv5* in the Schwann cell lineage disrupts cell function, leading to reduced myelination, sciatic nerve degeneration, and muscle atrophy. These deficits underscore *Etv5*'s essential role in maintaining nerve integrity.

## Introduction

**Figure 1.**
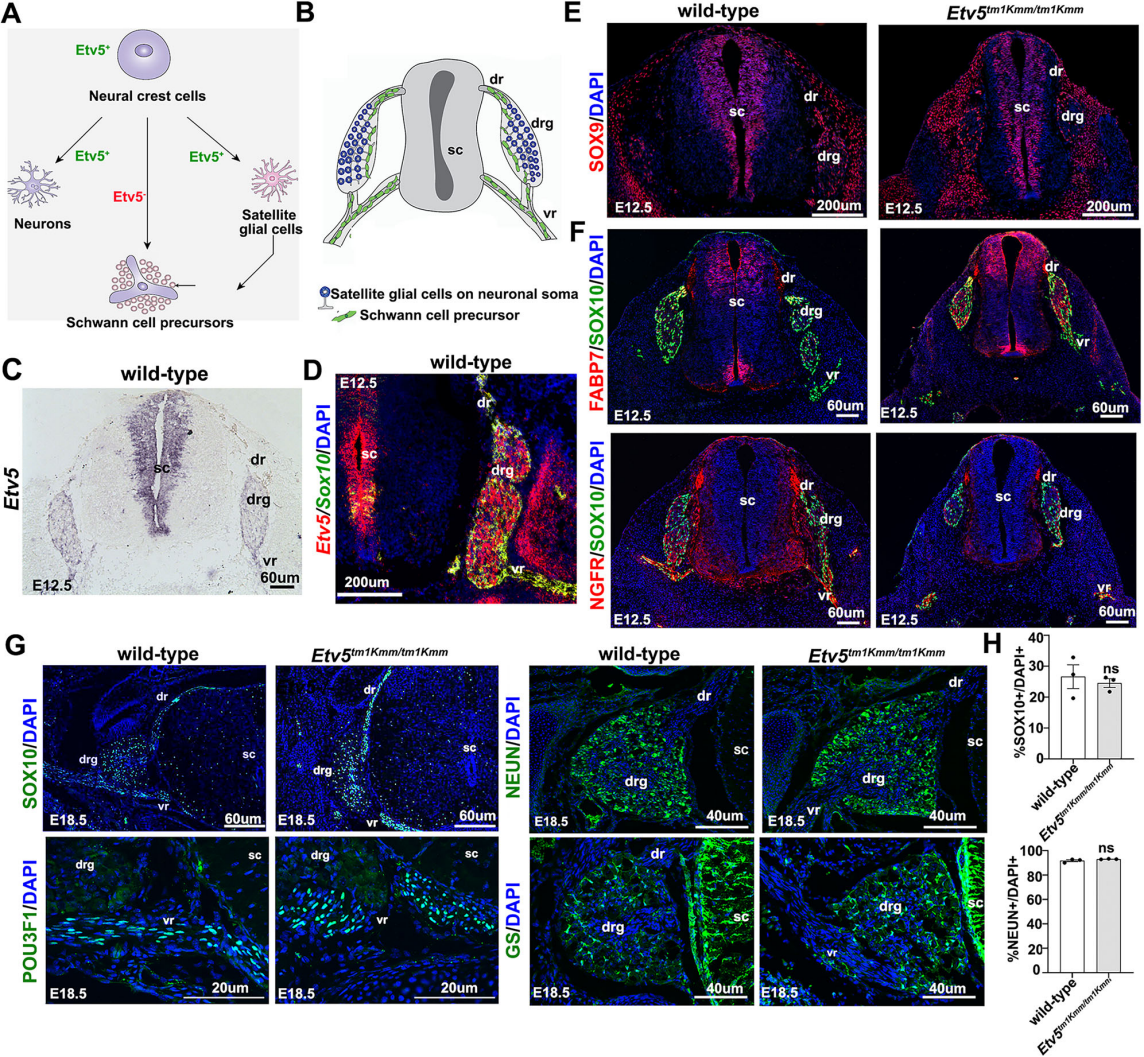
Schwann cell development occurs normally in embryos homozygous for an *Etv5^tm1Kmm^* hypomorphic allele. ***A***, Summary of *Etv5* expression during the transition of neural crest cells to sensory neurons, satellite glia, and Schwann cell precursors. ***B***, Schematic representation of E12.5 trunk section, showing satellite glia surrounding neuronal soma in the DRG, and Schwann cell precursors migrating into the ventral and dorsal roots and nerve trunk. ***C***, ***D***, Distribution of *Etv5* transcripts (***C***) and RNAscope of *Etv5* colocalized with *Sox10* transcripts (***D***) in transverse sections of E12.5 wild-type spinal cord, showing coexpression of *Etv5* (red) with *Sox10* (green) in ***D***. Blue is DAPI counterstain. ***E***, Expression of SOX9 in E12.5 wild-type and *Etv5^tm1Kmm^* homozygous transverse trunk sections. Blue is DAPI counterstain. Single channel images shown in grayscale below. Scale bars, 60 μm. ***F***, Coexpression of SOX10 (green) with FABP7 (red) or NGFR (red) in E12.5 wild-type and *Etv5^tm1Kmm^* homozygous transverse trunk sections. Blue is DAPI counterstain. Single channel images shown in grayscale below. Scale bars, 60 μm. ***G***, Labeling of SOX10, NEUN, POU3F1, and Glutamine Synthetase (GS) in E18.5 wild-type and *Etv5^tm1Kmm^* homozygous transverse sections through the lumbar spinal cord. Scale bars: SOX10, 60 μm; NEUN, GS, 40 µm; POU3F1, 20 µm. ***H***, Percentage of SOX10^+^/DAPI^+^ cells and NEUN^+^/DAPI^+^ cells in the E18.5 DRG (*N* = 3 animals/genotype). Graphs show means ± SEM. *p* values calculated with an unpaired *t* test. ns, nonsignificant; drg, dorsal root ganglion; sc, spinal cord; vr, ventral root. Extended Data Figure 1-1 supports this figure.

The two main macroglial cell types in the peripheral nervous system (PNS) are Schwann cells and satellite glia. Schwann cells wrap and myelinate peripheral nerve axons, while satellite glia encircle neuronal cell bodies in peripheral ganglia, including the dorsal root ganglia (DRG). Schwann cells and satellite glia both arise from neural crest cell (NCC) progenitors (Jessen and Mirsky, 2019; Fig. 1*A*). Consistent with their close lineage relationship, satellite glia can transition to a Schwann cell fate, at least in vitro (Cameron-Curry et al., 1993; Hagedorn et al., 2000), suggesting that satellite glia could replenish Schwann cells when required for repair. Schwann cells have two main functions: myelination and support of axons. Myelinating Schwann cells wrap around larger axons to form the myelin sheath, which speeds up nerve signal transmission, whereas non-myelinating Schwann cells surround smaller axons (Jessen and Mirsky, 2005). Axons require close association with Schwann cells to maintain axonal health and integrity (Gatzinsky et al., 2003; Li et al., 2018), resembling the role of oligodendrocytes in the central nervous system (CNS), another myelinating glial cell type (Fünfschilling et al., 2012; Lee et al., 2012; Morrison et al., 2013; Saab et al., 2013). In contrast, satellite glia are non-myelinating and instead maintain a homeostatic environment to modulate neuronal activity, functioning akin to CNS astrocytes. The role of satellite glia in regulating the microenvironment directly influences complex physiological responses, such as the pain response (Jasmin et al., 2010; Ji et al., 2013; Song et al., 2018; Wang et al., 2019; Zhang et al., 2020).

The ErbB family of receptor tyrosine kinases (RTKs), activated by Neuregulin 1 (NRG1), are required for Schwann cell differentiation and myelination (Newbern and Birchmeier, 2010). MEK-ERK1/2 signaling is activated by NRG1 and similarly plays a vital role in the Schwann cell lineage (Grossmann et al., 2009; Newbern et al., 2011; Sheean et al., 2014). Transient ERK signaling drives myelin disassembly during Wallerian degeneration, an early step in peripheral nerve repair (Ishii et al., 2013; Sheean et al., 2014). However, long-term ERK activation in Schwann cells negatively impacts nerve repair by hindering axon remyelination, the later stage of the injury response (Napoli et al., 2012; Cervellini et al., 2018). ERK kinases, specifically ERK1 and ERK2, phosphorylate various substrates, including Erythroblast Transformation Specific (Ets)-domain transcription factors (TFs; Yang et al., 2013). In Drosophila, *Pointed* (*Pnt*) is an Ets-domain TF that is activated downstream of RTK signaling and required for glial cell fate specification (Klaes et al., 1994). In vertebrates, Ets-domain TFs of the PEA3 family are activated by the MEK-ERK pathway, including *Etv1* (*ER81*), *Etv4* (*Pea3*), and *Etv5* (*Erm*). In the CNS, MEK-ERK signaling induces *Etv1* and *Etv5* expression to specify a myelinating oligodendroglial cell fate (Li et al., 2012, 2014; Wang et al., 2012; Ahmad et al., 2019). In the PNS, *Etv1* is expressed in Schwann cells, facilitating peripheral axon connections with non-myelinating Schwann cells, particularly in Pacinian corpuscles (Sedy et al., 2006; Srinivasan et al., 2007; Fleming et al., 2016). Additionally, *Etv1* regulates Schwann cell proliferation, maturation, and myelination (Askar et al., 2022). Comparatively, *Etv4* is understudied in PNS glia but is crucial for the development of sensory neurons involved in pain perception (Ríos et al., 2022). Finally, *Etv5* is expressed beginning at embryonic day (E) 9.0 in NCCs as well as in Schwann cell precursors (SCPs) and satellite glia until at least E12.5 (Hagedorn et al., 2000; Balakrishnan et al., 2016). Blocking *Etv5* function in NCCs hinders neuronal differentiation without impacting proliferation, glial differentiation, and survival in vitro (Paratore et al., 2002). Whether *Etv5* is required for Schwann cell development, axonal maintenance or nerve repair in vivo has yet to be addressed.

Animals homozygous for an *Etv5* null allele (*Etv5^tm1Hass^*) die by E8.5 (Chen et al., 2005; Kuure et al., 2010). Thus, to ask whether *Etv5* is required for Schwann cell development, we employed a hypomorphic *Etv5^tm1Kmm^* mutation, in which exons 2–5 were deleted (Chen et al., 2005). We also generated an *Etv5*-conditional knock-out (cKO) in the Schwann cell lineage using *Sox10-Cre*. In *Etv5^tm1Kmm^* homozygous embryos, Schwann cells develop normally. However, the normal expansion of Schwann cells in response to sciatic nerve injury is attenuated in *Etv5*-cKO male mice by 6 months of age, albeit without impacting motor function. Even in the absence of injury, the structural integrity of the sciatic nerve was impaired in *Etv5*-cKO male and female mice, and the gastrocnemius muscle, which is innervated by a branch of the sciatic nerve, was atrophic, with a delayed effect in female mice. Thus, *Etv5* is required to maintain the structural integrity of peripheral nerves and the health of the muscles that the sciatic nerve innervates.

## Materials and Methods

### Animals and genotyping

Animal procedures were approved first by the University of Calgary and later by the Sunnybrook Research Institute Animal Care Committees (AUP 20-606), in compliance with the Guidelines of the Canadian Council of Animal Care. *Etv5^tm1Kmm^*/J mice (Stock No. 022300), Rosa-tdTomato (Stock No. 007914), *Sox10-Cre* (Stock No. 025807), and *Etv5*^fl/fl^ mice (Stock No. 025534) mice were obtained from Jackson Laboratory. *Etv5^tm1Kmm^*/J mice were maintained on a 129/SvJ background (Charles River 287) and *Etv5*-cKO mice were maintained on a C57BL/6J background (Strain No. 000664, Jackson Laboratory). CD1 mice were used for RNAscope (Charles River 022). All mice were maintained in a 12 h light/dark cycle. Pregnancy was determined by detection of a vaginal plug, with the morning of plugging designated as E0.5. Analyses were performed on male and female animals at postnatal stages after postnatal day (P) 0 and on embryos of both sexes up until P0. PCR genotyping was performed with the following primers: wild-type forward primer: TCT GGC TCA CGA TTC TGA AG; *Etv5^tm1Kmm^*/J mutant forward primer: AAG GTG GCT ACA CAG GCA AG and common reverse primer: CGG AGG TCA AGC TGT TAA GG. PCR primers for *Etv5*-cKO genotyping included the following: *Etv5*-forward: GCC TAC AGT GAT TCC GTG GT; *Etv5*-reverse: TGT GCA CAT GTT CAA GG; *Sox10-Cre*-forward: CCT GCT GGA AGA TGG CGA TTA G; *Sox10-Cre* reverse: TGA AGA GGG GGC GGA GAA AG. For the *Rosa-tdTomato* allele, PCR primers included the following: wild-type *Rosa* locus-forward: AAG GGA GCT GCA GTG GAG TA; and reverse: CCG AAA ATC TGT GGG AAG TC; and recombined *Rosa-tdTomato* locus-forward: CTG TTC CTG TAC GGC ATG G and reverse: GGC ATT AAA GCA GCG TAT CC.

### Embryo collection

Embryo trunks or postnatal nerves were dissected in ice-cold phosphate-buffered saline (PBS) and then fixed overnight in 4% paraformaldehyde (PFA)/PBS. Fixed tissue was washed in PBS, immersed in 20% sucrose/PBS overnight, and then blocked in O.C.T. (Tissue-Tek, Sakura Finetek USA) before storing at −80°C. Blocked tissue was sectioned on a Leica cm3050s cryostat at 10 µm and collected on SuperFrost Plus slides (Thermo Scientific).

### Peripheral nerve crush

Peripheral nerve crush injuries were performed on the sciatic nerve of P21–30 or 6-month-old wild-type and *Etv5* cKO mice as previously described (Balakrishnan et al., 2016). Briefly, P21–30 or 6-month-old male and female animals were anesthetized (5% isoflurane for induction and 2% for maintenance), hindlimbs were shaved and sterilized, and a small incision was made to expose the sciatic nerve. A crush injury was performed using #10 forceps for 30 s, marking the site of injury with activated charcoal sterilized under UV light for 2 h prior, and then the muscle and skin were sutured back together (Bauder and Ferguson, 2012). Body temperature was maintained using a heating pad throughout the procedure. Buprenorphine subcutaneous injection of 0.1 ml (100 μl of 0.03 mg/ml) was administered for pain on the day of surgery and for 3 d following, with the nerve harvested on day 5.

### Behavioral assessment

Animals (*N* = 5 of both sexes for each genotype) were handled for 3 consecutive days for 10 min each, followed 1–2 d later by baseline gait analysis on a CatWalk (Noldus Information Technology) version 11 system. Mice walked along a transparent corridor toward the home cage and their footprints were analyzed with a high frame rate camera and red and green light sources that allow the pawprints to be visualized with increased contrast. One to two days after handling, a sciatic nerve crush injury was performed, designated as Day 0, followed by endpoint gait analysis 5 d later. Baseline runs were conducted, recording footprints with Noldus system software, of which at least three runs with a maximum speed variation of 60% were selected for analysis. Using recorded footprints from the right hind (RH) limb and normalized to baseline values, the change in standing time, print area, swing time, and print intensity between Day 0 (baseline) and Day 5 was evaluated. To quantify changes between Day 0 and Day 5, area under the curve (AUC) measurements were calculated. In addition, on each day between Days 0 and 5, a toe spread analysis was performed. A fully clasped paw was assigned a score of 0, a paw with intermediate toe spread was assigned a score of 1, and a fully extended toe spread was assigned a score of 2.

### Ultrasound (US) imaging

Male and female animals (*N* = 3 of either sex) were anesthetized using isoflurane (4% induction, 2% maintenance, and 1 L/min oxygen), and the body temperature was maintained using a heating pad during the imaging session. All animal experimental procedures were approved by the Animal Care Committee at Sunnybrook Research Institute. Prior to ultrasound acquisitions, the imaging area was carefully shaved and depilatory cream was applied for complete fur removal. Furthermore, eye lubricant was placed on each eye to prevent drying during the imaging process. Ultrasound imaging was performed at two different time points (2 and 6 months of age), investigating the downstream effect due to nerve injury (*N* = 3 each for control and mutant groups). The mouse was prone positioned, and the scanning side lower limb was fixed by placing either gauze or a food pellet under the femur to enlarge the contact surface. For gastrocnemius muscle cross-sectional area (CSA) and volume evaluations, ultrasound imaging was performed on the mice using a Vevo 3100 (FUJIFILM VisualSonics) system and a MX400 linear array transducer (bandwidths of 20–46 MHz with 30 MHz center transmit frequency and 50 µm axial resolution). Dynamic range was set to 60 dB and the acquisition gain was set to 25 dB for all acquisitions. For volumetric imaging, the 3D range is kept to a maximum of ∼16 mm with a step size of 0.152 mm per frame covering the entire mouse tibia from proximal to distal end using a linear motor stage. For consistency, the largest CSA measurement of each muscle is used as the reference center midpoint. For volume measurement, four frames proximal and distal from the midpoint (total of nine frames) are used for volume calculation. Commercial Vevo Lab software version 5.10.0 was used to calculate both CSA and volume of the acquired US images.

### Immunohistochemistry

Sections were thawed, rinsed in PBS to remove excess O.C.T., permeabilized in PBT (PBS with 0.1% Triton X), and then blocked in 10% normal horse serum/PBT for 1 h. Primary antibodies were then diluted in blocking solution and incubated on sections overnight at 4°C, followed by three PBT washes. Species-specific secondary antibodies, conjugated to Alexa 488 or Alexa 555, were diluted 1/500 in PBT and applied to sections for 1 h. Sections were washed three times in PBT and stained with 4′,6-diamidino-2-phenylindole (DAPI; Santa Cruz Biotechnology; 1:5,000 in PBT). Sections were washed three times in PBS and mounted in AquaPolymount (Polysciences). Primary antibodies included the following: rabbit anti-ETV5 (Abcam ab102010; 1:300), rabbit anti-TFAP2A (Abcam; ab52222; 1:200), goat anti-OCT6 C-20 (Santa Cruz Biotechnology; sc-11661; 1:50), rabbit anti-SOX9 (Millipore; AB5535; 1:500), goat anti-SOX10 (Santa Cruz Biotechnology; sc-17343; 1:400), rabbit anti-SOX10 (Abcam; AB227680; 1:200), mouse anti-SOX10 (Proteintech 66786; 1:200), rabbit anti-GFAP (Dako Cytomation; #Z0334; 1:500), rabbit anti-NGFR (Millipore; #07-476;1:500), rabbit anti-FABP7 (Millipore; ABN14; 1:500), rabbit anti-JUN (Cell Signaling Technology 9165, 1:400), and mouse anti-NEUN (Millipore MAB377; 1:200). For anti-rat KI67 (Invitrogen 14-5698-82; 1:250), antigen retrieval treatment was performed using 100 mM citrate buffer, pH 6, in a microwave with power setting 3 for 1 min before primary staining. FluoroMyelin (Life Technologies F34651) was diluted in PBT at 1:50 and incubated on the slides for 30 min before washing in PBS, counterstaining with DAPI, and mounting in AquaPolymount.

### RNA in situ and RNAscope hybridization

A digoxigenin-labeled *Etv5* riboprobe was generated as previously described using a 10× labeling mix and following the manufacturer's instructions (Roche; Li et al., 2014). The probe was hybridized overnight, and washing and staining procedures were followed as previously described (Touahri et al., 2015). RNAscope was performed using a Multiplex Fluorescent Detection Kit v2 (ACD; #323110) according to the manufacturer's instructions. ACD probes used included the following: Mm-*Sox10*-C2 (catalog #435931) and Mm-*Etv5*-C1 (catalog #316961). Hybridized transcripts were detected using Opal 570 (Akoya; #FP1488001KT; 1:1,500) for channel 1 probes and Opal 520 (Akoya; #FP1487001KT; 1:1,500) for channel 2 probes. Posthybridization and detection, sections were counterstained with DAPI and mounted in AquaPolymount.

### Transmission electron microscopy

Prior to sciatic nerve dissection, male and female animals at P30 and 6 months of age were anesthetized using ketamine (75 mg/kg, Narketan, 0237499) and xylazine (10 mg/kg, Rompun, 02169592). Animals were then perfused intracardially using a peristaltic pump at 10 ml/min, first delivering a 20-fold blood volume of ice-cold saline (0.9% NaCl, Braun, L8001). Next, animals were perfused with 2% paraformaldehyde (PFA, Electron Microscopy Sciences, catalog #19208) and 2% glutaraldehyde (Electron Microscopy Sciences, catalog #12300) diluted in 0.1 M phosphate buffer for 5 min, as described (Ferri et al., 2018). Nerves of each genotype and stage were stored in this mixture and taken to and performed by the Cellular and Molecular Electron Microscopy (CMEM) facility at The Hospital for Sick Children (Toronto) for processing and semithin sectioning. Briefly, perfused nerves were immersed in 0.1 M sodium cadocylate (Electron Microscopy Sciences, catalog #243 12300) in PBS for 1 d at 4°C before postfixation in 1% OsO4 (Electron Microscopy Sciences, catalog #19152) for 90 min. Nerves were washed in buffer for 20 min and then taken through a dehydration protocol in graded ethanol steps for 20 min each, at a step of 10% from 50 to 100%. Tissues were infiltrated with propylene oxide (PPO; Electron Microscopy Sciences, catalog #20412) for 1 h, and then PPO was gradually replaced with a Spur resin over 2 h. Resin infiltration continues overnight in fresh resin. The following day, nerves were embedded in Flat silicon molds and cured in the oven at 65°C for 1 d. To examine the ultrastructure of the sciatic nerve, transverse sections were imaged using a Hitachi HT7800 transmission electron microscope Hedwig microscope and an EMSIS Xarosa 20 Megapixel CMOS camera. Three representative images were chosen from each replicate and quantified using MyelTracer software (Kaiser et al., 2021).

### scRNA data mining

Single-cell RNA-sequencing data was mined from Lovatt et al. (2022) (Accession: GSE216665) and reprocessed using Seurat v.4.3.0 R package (Hao et al., 2021). The data was transformed by the SCTransform function while regressing out the variance due to mitochondrial RNAs. Batch effect was corrected using Harmony (Korsunsky et al., 2019). Clustering was performed by the RunPCA, FindNeighbors, and FindClusters functions using the first 30 principal components. The 2D projection of the clustering was carried out by the RunUMAP function. Gene expression was visualized using the DotPlot and FeaturePlot functions.

### Sanger sequencing

PCR genotyping products were run out on a 1.5% low melting point agarose gel and the mutant band was cut out using QIAquick Gel Extraction Kit (Qiagen, 28704). The DNA was purified and sent for Sanger sequencing at the TCAG DNA Sequencing Facility at The Hospital for Sick Children (Toronto) using the following primers: *Etv5*-forward: TGT GCT TCT GCT GTA GCC CG; *Etv5*-reverse: TCT CAA CCT TGT GGA CGA CC.

### Digital droplet PCR (ddPCR)

DNA was extracted from nerves and purified. QX200 droplet digital PCR system (Bio-Rad) was used to quantify the copy number of the *Etv5* mutant allele, using a probe specific to the recombined *Etv5*^fl^ locus. To detect the *Etv5* mutant allele, forward primer 5′-GGA ACT TCA TCA GTC AGG TAC A-3′, reverse primer 5′-CCA CCA CCA AAC TCG GAT-3′, and probe 5′-6-FAM-TGC TAT ACG -ZEN-AAG TTA TTA GGT CCC TCG AGG -Iowa Black FQ-3′ (Integrated DNA Technologies) sequences were used. The *ApoB* forward primer 5′-CGT GGG CTC CAG CAT TCT A-3′, reverse primer 5′-TCA CCA GTC ATT TCT GCC TT TG-3′, and probe 5′-HEX-CCT TGA GCA-ZEN-GTG CCC GAC CAT TG- Iowa Black FQ-3′ (Integrated DNA Technologies) sequences were used to normalize *Etv5* gene copies per cell. The ddPCR reaction was performed in a 20 μl volume containing 10 μl of 2X QX200 ddPCR Supermix for Probes (No dUP; catalog #1863023, Bio-Rad), 10 ng of genomic DNA, 900 nM of the forward and reverse *Etv5* primers, 250 nM of *Etv5* probes, 900 nM of the forward and reverse *ApoB* primers, 250 nM *ApoB* probe. Each ddPCR assay mixture was loaded into a disposable droplet generator cartridge (catalog #1864008, Bio-Rad). Then, 70 μl of droplet generation oil for probes (catalog #1863005, Bio-Rad) was loaded into each of the eight oil wells. The cartridge was then placed inside the QX200 droplet generator (Bio-Rad). When droplet generation was completed, the droplets were transferred to a ddPCR 96-well plate (catalog #12001925, Bio-Rad) using multichannel pipet. The plate was heat-sealed with foil and placed in C1000 Touch Thermal Cycler (Bio-Rad). Thermal cycling conditions were as follows: 95°C for 10 min, then 44 cycles of 94°C for 30 s and 60°C for 1 min, followed by 98°C for 10 min, and a 4°C indefinite hold. FAM fluorescent signal for *Etv5* DNA sequence and HEX fluorescent signal for the *ApoB* gene sequence in each droplet were counted using a QX200 digital droplet reader and analyzed by QuantaSoft analysis software ver.1.7.4.0917 (Bio-Rad). All ddPCR analyses were performed at the Sunnybrook Research Institute Genomics Core Facility.

### Microscopy and image analysis

Images were captured with a QImaging RETIGA EX digital camera and a Leica DMRXA2 optical microscope using OpenLab5 software (Improvision), or with a Nikon A1 confocal. Image processing and analysis were performed using ImageJ software. Three images per wild-type and *Etv5^tm1Kmm^* homozygous embryo/nerve were assessed. DAPI channel images were converted into 8 bit format and the threshold was set using weighted mean intensity. Images were inverted for binary conversion. In E18.5 sections, the dorsal root ganglionic region was manually selected using a free-form selection tool. The number of DAPI^+^ cells in the selected area was calculated using the particle analysis option. The colocalization of DAPI with the green and/or red channel (SOX10^+^/NEUN^+^ cells, SOX10^+^/KI67^+^) was calculated manually using Adobe Photoshop software in a selected region of interest. The % area of FluoroMyelin was calculated with a default threshold set in a selected region of interest.

### Statistical analysis

A minimum of three biological replicates were carried out for all assays. Statistical analysis and graphs were generated using GraphPad Prism 10.5.0 software. Student's *t* test (when comparing two groups) or one-way ANOVA with Tukey’s post hoc corrections (when comparing groups of more than two) were used. Repeated-measures ANOVA was used for behavior. All data expressed as mean value ± standard error of the mean (SEM). In all experiments, a *p* value <0.05 was considered statistically significant.

### Figure preparation

Sections were imaged on a Leica DMRXA2 optical microscope (Leica Microsystems Canada) using LasX software or using a Leica SP8 spectral confocal microscope (Leica Microsystems CMS). Composite figure were produced using Adobe Photoshop 26.8 (Adobe Systems) and a license from BioRender.com was used to prepare schematics.

## Results

### Schwann cell precursors develop normally in peripheral ganglia in mice homozygous for an *Etv5^tm1Kmm^* hypomorphic allele

*Etv5* is expressed in NCCs as early as E9.0 and later is expressed in NCC derivatives, including neuronal and Schwann cell lineage cells, persisting until at least E12.5 (Balakrishnan et al., 2016; Hagedorn et al., 2000; Fig. 1*A*,*B*). To confirm these findings, we performed RNA in situ hybridization on transverse sections through the E12.5 trunk, showing a wide distribution of *Etv5* transcripts in the DRG and in the spinal cord ventricular zone in wild-type embryos (Fig. 1*C*). To determine whether *Etv5* was expressed in the Schwann cell lineage, we performed RNAscope in situ hybridization using probes for *Etv5* and *Sox10*, a myelinating glial lineage marker (Fig. 1*D*). A subset of *Etv5*^+^ cells coexpressed *Sox10* in the PNS, including Schwann cells and satellite glia in E12.5 DRGs, and Schwann cells in the ventral and dorsal roots. Of note, *Etv5* was also expressed in *Sox10*-negative cells, likely corresponding to NCC-derived DRG sensory neurons (Fig. 1*D*).

10.1523/ENEURO.0410-20.2025.f1-1 Figure 1-1 **Schwann cell lineage markers are expressed normally in immature Schwann cells in E15.5 *Etv5^tm1Kmm^* homozygous mutant embryos.** **1-1(A)** Low magnification DAPI-stained images of transverse sections through the lumbar spinal cord of E15.5 wild-type and *Etv5^tm1Kmm^* homozygous embryos. Scale bars, 100 μm. **1-1(B-D)** Expression of SOX9 (B), SOX10 (C) and TFAP2A (D) in transverse sections through the lumbar spinal cord of E15.5 wild-type and *Etv5^tm1Kmm^* homozygous mutant embryos. Scale bars, 60 μm. **1-1(E-H)** Co-expression of SOX10 (green) with SOX2 (red, E), FABP7 (red or black/white, F), GFAP (red or black/white, G), and NGFR (red or black/white, H), counterstained with DAPI (blue) in transverse sections through the lumbar spinal cord of E15.5 wild-type and *Etv5^tm1Kmm^* homozygous mutant embryos. dr, dorsal root; drg, dorsal root ganglion; sc, spinal cord; vr, ventral root. Scale bars (A,B), 100 μm; (C-H), 60 μm. Download Figure 1-1, TIF file.

To assess whether *Etv5* is required for Schwann cell development, we employed *Etv5^tm1Kmm^* homozygous mutant embryos, in which exons 2–5 were deleted, encoding for the initiation codon and transactivation domain. SOX9, a marker of the glial lineage beginning at the NCC stage (Cheung and Briscoe, 2003; Balakrishnan et al., 2016), was expressed in the DRG, ventral root, neural tube, and trunk mesenchymal cells in embryos of both genotypes at E12.5 (Fig. 1*E*). To assess Schwann cell maturation, we examined the coexpression of SOX10 with Fatty Acid-binding Protein 7 (FABP7), which is expressed in SCPs in a *Sox10-*dependent manner (Finzsch et al., 2010), and with Nerve Growth Factor Receptor (NGFR, or p75NTR), the deletion of which reduces peripheral ganglia size and Schwann cell number (von Schack et al., 2001). Both FABP7 and NGFR were similarly expressed in SOX10^+^ cells in the DRG and dorsal and ventral roots in E12.5 wild-type and *Etv5^tm1Kmm^* homozygous embryos (Fig. 1*F*).

By E15.5, SCPs differentiate into immature Schwann cells that populate developing spinal ganglia and nerves (Jessen and Mirsky, 2005). In the lumbar spinal cord of E15.5 wild-type and *Etv5^tm1Kmm^* homozygous embryos, SOX9 and SOX10 were similarly expressed in scattered SCPs and immature Schwann cells throughout the DRG, as well as in the dorsal and ventral roots (Extended Data Fig. 1*A–C*). We also examined the expression of two TFs with a later onset of expression in SCPs, including TFAP2A (Stewart et al., 2001; Balakrishnan et al., 2016) and SOX2, an inhibitor of Schwann cell myelination expressed in SCPs and immature Schwann cells only (Le et al., 2005; Adameyko et al., 2012; Balakrishnan et al., 2016). In both E15.5 wild-type and *Etv5^tm1Kmm^* homozygous DRGs, TFAP2A was widely expressed, while SOX2 was only detected in a few SOX10*^+^* Schwann cells (Extended Data Fig. 1*D,E*). Finally, to assess the maturation process of SCPs into immature Schwann cells, we examined the expression of FABP7, glial fibrillary acidic protein (GFAP) and NGFR (Extended Data Fig. 1*F–H*). All three of these proteins were expressed in scattered SOX10^+^ SCPs and immature Schwann cells in E15.5 DRGs of both genotypes (Extended Data Fig. 1*F–H*).

By E18.5, a subset of immature Schwann cells begin to associate with large-diameter axons to become promyelinating Schwann cells, whereas immature Schwann cells in contact with smaller-diameter axons become non-myelinating Schwann cells (Feltri et al., 2015). In transverse trunk sections of E18.5 wild-type and *Etv5^tm1Kmm^* homozygous embryos, similar numbers of SOX10^+^ Schwann cells were detected in the DRG, as well as the dorsal and ventral roots (Fig. 1*G*,*H*). Additionally, a similar number of NEUN^+^ neurons were observed in wild-type and *Etv5^tm1Kmm^* homozygous mutant DRGs (Fig. 1*G*,*H*), indicating that sensory neurons are produced in normal quantities even in the absence of *Etv5*. Next, we examined the expression of POU3F1 (OCT6), which is expressed in late immature and promyelinating Schwann cells and is required for the transition to a myelinating phenotype (Arroyo et al., 1998). POU3F1 expression was detected in the ventral roots in both E18.5 wild-type and *Etv5^tm1Kmm^* homozygous embryos, suggesting that SOX10 cells mature to a myelinating stage in the absence of *Etv5* (Fig. 1*G*). Finally, to detect satellite glial cells in the developing DRG, we examined the expression of glutamine synthetase (GS; Ohara et al., 2009), revealing that this marker was expressed comparably in both wild-type and *Etv5^tm1Kmm^* homozygous mutant DRGs (Fig. 1*G*). Thus, Schwann cell and satellite glial differentiation and maturation are not grossly perturbed in *Etv5^tm1Kmm^* homozygous embryos.

### Generation of a Schwann cell-specific *Etv5* conditional knock-out (cKO)

**Figure 2.**
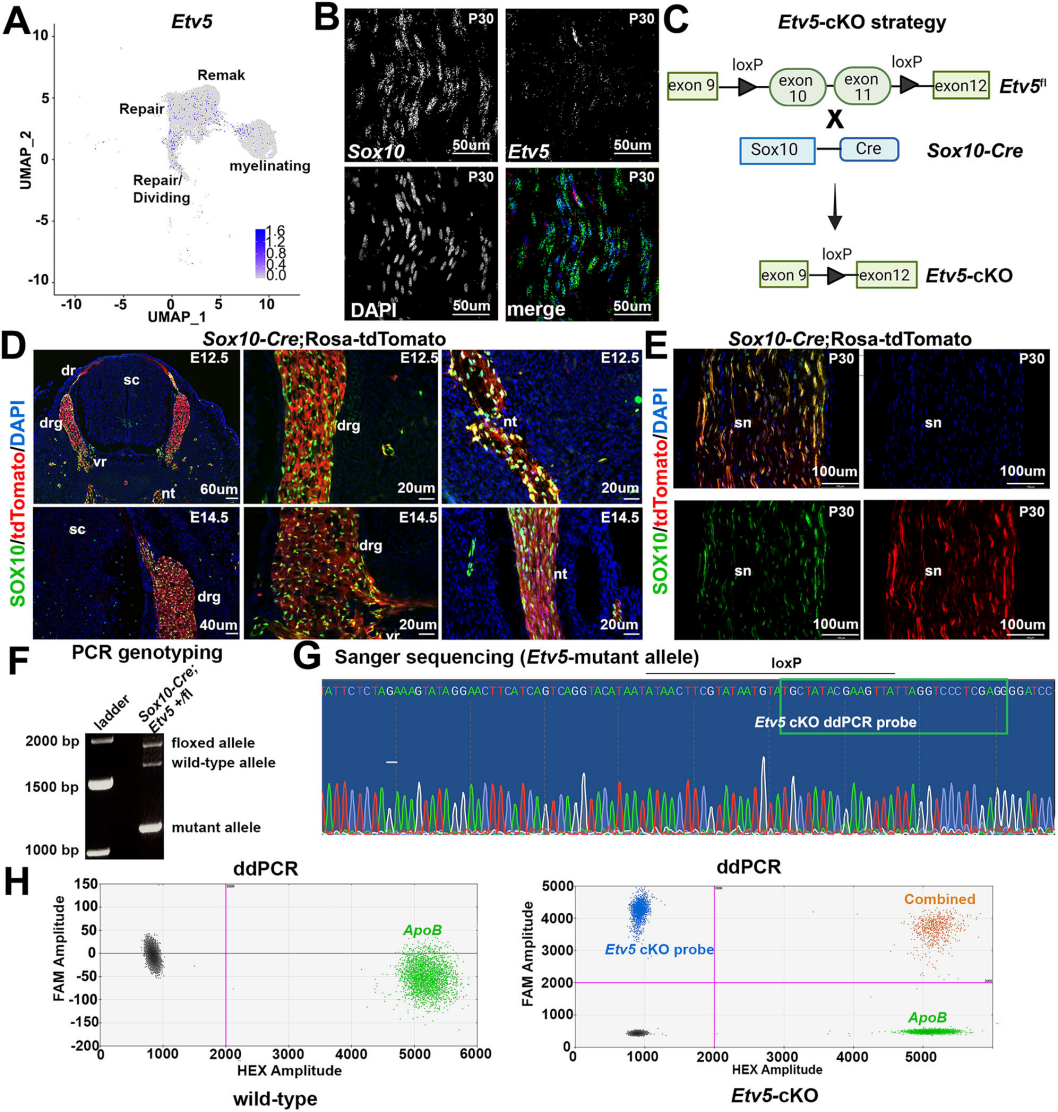
Generation of a Schwann cell-specific *Etv5* conditional knock-out. ***A***, Feature plot showing *Etv5* transcript distribution in Remak, repair, repair/dividing, and myelinating Schwann cells, in mined single-cell RNA-sequencing data (Lovatt et al., 2022; Accession: GSE216665). ***B***, RNAscope hybridization of P30 sciatic nerve with probes for *Etv5* (red) and *Sox10* (green). Blue is DAPI counterstain. Scale bars, 50 μm. ***C***, Schematic illustration of *Etv5*-cKO strategy (prepared with a license from BioRender.com). ***D***, ***E***, Lineage trace using *Sox10-Cre* using a *Rosa-tdTomato* reporter, showing tdTomato expression in SOX10^+^ cells in the DRG and Schwann cells in the dorsal root and ventral root at E12.5 (***D***) and in Schwann cells in the sciatic nerve at P30 (***E***). Scale bars in ***D***, 60 μm (top left), 40 μm (bottom left), 20 μm (middle, right top and bottom); in ***E***, 100 μm. ***F***, PCR genotyping of nerve collected from *Sox10-Cre;Etv5*^fl^ mice, showing wild-type and floxed *Etv5* allele, and the recombined “mutant” *Etv5* allele after Cre-mediated recombination. ***G***, Sanger sequencing of the recombined “mutant” *Etv5* allele, showing the position of the probe used for ddPCR that was specific to the recombined allele. ***H***, ddPCR of P30 wild-type and recombined *Etv5*-cKO sciatic nerves, showing a positive signal for the *Etv5* recombined “mutant” allele only in *Etv5*-cKO nerves (blue). An *ApoB* control probe was used to normalize *Etv5* gene copies per cell (green). The signal for droplets containing both wild-type and “mutant” targets was also only present in *Etv5*-cKO nerves (orange). Negative droplets containing no targets (gray).

We next sought to evaluate *Etv5* function in mature Schwann cells at postnatal stages. We first asked whether *Etv5* expression persisted in the postnatal peripheral nerve by mining single-cell (sc) RNA-sequencing data from naive and injured nerves collected at 3, 12, and 60 d post-injury (Lovatt et al., 2022). We constructed a high-dimensional plot of the sequencing data by generating uniform manifold approximation and projection (UMAP) plots, in which we subclustered the region containing myelinating, Remak (non-myelinating), repair, and repair/dividing Schwann cells. In a feature plot, *Etv5* transcripts were detected at low levels in Schwann cells in each state (Fig. 2*A*). Finally, we confirmed *Etv5* is expressed in a subset of *Sox10*^+^ Schwann cells in the P30 peripheral nerve by RNAscope hybridization (Fig. 2*B*).

Since the *Etv5^tm1Kmm^* mutation is considered a hypomorphic allele rather than a null mutation, we generated conditional knock-out (cKO) mice to examine the role of *Etv5* at postnatal stages. To generate *Etv5*-cKOs, we crossed mice carrying a floxed *Etv5* allele in which the loxP sites flanked exons 10–11, encoding part of the DNA binding domain (Zhang et al., 2009), with a glial-specific *Sox10-Cre* driver line (Stine et al., 2009; Fig. 2*C*). We used a *Rosa-tdTomato* reporter to confirm that *Sox10-Cre* induced recombination in Schwann cells. At E12.5, tdTomato was coexpressed with SOX10 in the dorsal and ventral roots, nerve trunk and DRG (Fig. 2*D*). *Sox10-Cre* also recombined the *Rosa-tdTomato* reporter allele in SOX10^+^ Schwann cells in the P30 peripheral nerve (Fig. 2*E*).

Since Cre drivers can display differences in recombination efficiency depending on the floxed locus, we set out to ensure that the *Etv5*^fl^ allele was efficiently targeted by *Sox10-Cre*. Since the *Etv5*^fl^ allele was not fully annotated, we first conducted PCR genotyping of P30 *Sox10-Cre;Etv5*^fl/+^ nerves, revealing three bands in order of size: a floxed allele, a non-modified wild-type allele, and a mutant allele. To confirm the mutant allele corresponded to the *Etv5* locus, the band was cut out of the gel and subjected to Sanger sequencing (Fig. 2*F*), revealing the position of the remaining loxP site in the *Etv5* locus post-Cre-mediated recombination (Fig. 2*G*). We used this information to design an *Etv5* probe that would specifically label the recombined *Etv5*-cKO allele. Using digital droplet PCR (ddPCR), we confirmed that the *Etv5*^fl^ locus was successfully recombined in *Etv5*-cKO peripheral nerves (Fig. 2*H*).

### Schwann cell response to sciatic nerve crush is impaired in adult *Etv5*-cKO mice

**Figure 3.**
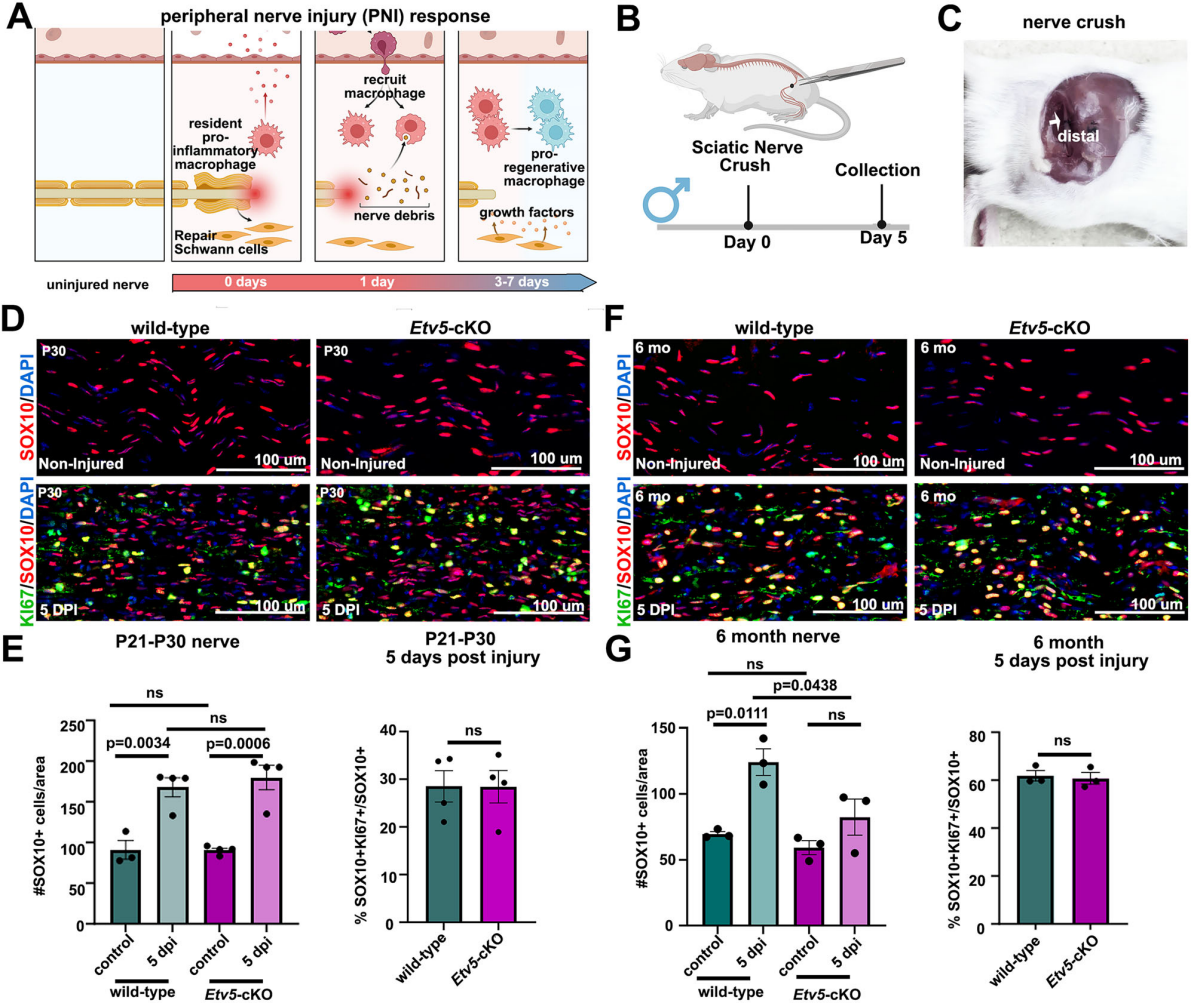
Defects in the response of male *Etv5*-cKO mice to a sciatic nerve crush. ***A***, Schematic representation of the peripheral nerve injury response (BioRender.com). ***B***, ***C***, Sciatic nerve crush was performed on male mice on day 0 and nerves were collected on Day 5 (BioRender.com; ***B***). Activated charcoal was used to mark the site of the crush (white arrow), so that the nerve analysis could be centered on sites distal to the crush site (***C***). ***D***, SOX10 (red) and KI67 (green) labeling of noninjured P21–30 nerves and at 5 d postinjury (dpi) collected from wild-type and *Etv5*-cKO male mice. Blue is DAPI counterstain. Scale bars, 100 μm. ***E***, Quantification of the percentage of SOX10^+^ cells/area in control, uninjured P21–P30 nerves, and at 5 dpi, collected from wild-type (*N* = 3 uninjured and *N* = 4 injured) and *Etv5*-cKO (*N* = 4 each) male mice. *p* values calculated with one-way ANOVA and post hoc Tukey’s test. Quantification of the percentage of SOX10^+^ cells that are proliferating (KI67^+^) in control, uninjured P21–P30 nerves, and at 5 dpi, collected from wild-type (*N* = 4) and *Etv5*-cKO (*N* = 4) male mice. *p* values calculated with an unpaired *t* test. ns, nonsignificant. ***F***, SOX10 (red) and KI67 (green) labeling of noninjured 6-month-old nerves, and at 5 d postinjury (dpi) collected from wild-type and *Etv5*-cKO male mice. Blue is DAPI counterstain. Scale bars, 100 μm. ***G***, Quantification of the percentage of SOX10^+^ cells/area in control, uninjured 6-month-old nerves, and at 5 dpi, collected from wild-type (*N* = 3) and *Etv5*-cKO (*N* = 3) male mice. *p* values calculated with an unpaired *t* test. Quantification of the percentage of SOX10^+^ cells that are proliferating (KI67^+^) in control, uninjured 6-month-old nerves, and at 5 dpi, collected from wild-type (*N* = 3) and *Etv5*-cKO (*N* = 3) male mice. *p* values calculated with one-way ANOVA and post hoc Tukey’s test. ns, nonsignificant. Extended Data Figures 3-1 and 3-2 support this figure.

After peripheral nerve injury, Wallerian degeneration begins, in which damaged axons distal to the lesion and their myelin sheaths degenerate. This degeneration is followed by a coordinated response from Schwann cells, which initiate proliferation 3–4 d post-injury (Yang et al., 2008) and switch to a repair phenotype, secreting growth factors to stimulate axon regeneration, downregulating myelin genes that hinder repair, and collaborating with macrophages to clear debris (Fig. 3*A*; Mirsky et al., 2008; Balakrishnan et al., 2020). To determine whether *Etv5* is required for the Schwann cell injury response, we applied a sciatic nerve crush to wild-type and *Etv5*-cKO mice, labeling the crush site with activated charcoal so that the Schwann cell repair response could be monitored distal to the injury (Fig. 3*B*,*C*; Extended Data Fig. 3-1*A*). A sciatic nerve crush was first generated in juvenile mice between P21 and P30. Nerves were collected at 5 d post-injury (dpi), at a time when most Schwann cells have dedifferentiated into a repair phenotype and begun to proliferate (Balakrishnan et al., 2016). In uninjured P21–P30 male (Fig. 3*D*,*E*) and female (Extended Data Fig. 3-1*B,C*) wild-type and *Etv5*-cKO mice, SOX10^+^ Schwann cells were scattered throughout longitudinal sections of the sciatic nerve and present in equivalent numbers in both genotypes. At 5 d post-injury, the number of SOX10^+^ Schwann cells increased in wild-type and *Etv5*-cKO male (Fig. 3*D*,*E*) and female (Extended Data Fig. 3-1*B,C*) nerves compared with the uninjured nerve. This increase was due to the proliferation of a subset of SOX10^+^ Schwann cells, as shown by colabeling with KI67. Notably, the Schwann cell proliferative fraction was equivalent in wild-type and *Etv5*-cKO male nerves at P21–P30 (Fig. 3*D*,*E*) and 6 months of age (Fig. 3*F*,*G*). In contrast, there was a transient increase in the Schwann cell proliferative fraction in *Etv5*-cKO female nerves (Extended Data Fig. 3-1*B–E*).

10.1523/ENEURO.0410-20.2025.f3-1 Figure 3-1 **Defects in the response of female *Etv5*-cKO mice to a sciatic nerve crush.** **3-1(A)** Sciatic nerve crush was performed on female mice on day 0 and nerves were collected on day 5 (BioRender.com). **3-1**(**B-C**) SOX10 (red) and KI67 (green) labeling of non-injured P 21-30 nerves and at 5-day post-injury (dpi) collected from wild-type and *Etv5*-cKO female mice. Blue is DAPI counterstain. Scale bars, 100 μm (B). Quantification of the percentage of SOX10^+^ cells/area in control, uninjured P21-30 nerves, and at 5  dpi, collected from wild-type (N = 4) and *Etv5*- cKO (N = 4) female mice (C). P-values calculated with one-way ANOVA and post-hoc Tukey test. Quantification of the percentage of SOX10^+^ cells that are proliferating (KI67^+^) in control, uninjured P21-30 nerves, and at 5  dpi, collected from wild-type (N = 4) and *Etv5*-cKO (N = 4) female mice. P-values calculated with an unpaired t-test. ns = non-significant (C). **3-1**(**D-E**) SOX10 (red) and KI67 (green) labeling of non-injured 6-month-old nerves, and at 5-day post-injury (dpi) collected from wild-type and *Etv5*-cKO female mice. Blue is DAPI counterstain. Scale bars, 100 μm (D). Quantification of the percentage of SOX10^+^ cells/area in control, uninjured 6-month-old nerves, and at 5  dpi, collected from wild-type (N = 3) and *Etv5*-cKO (N = 3) female mice. P-values calculated with one-way ANOVA and post-hoc Tukey test. Quantification of the percentage of SOX10^+^ cells that are proliferating (KI67^+^) in control, uninjured 6-month-old nerves, and at 5  dpi, collected from wild-type (N = 3) and *Etv5*-cKO (N = 3) female mice. P-values calculated with one-way ANOVA and post-hoc Tukey test. ns = non-significant (E). Download Figure 3-1, TIF file.

10.1523/ENEURO.0410-20.2025.f3-2 Figure 3-2 **JUN is expressed normally in repair Schwann cells in *Etv5-* cKO sciatic nerves at 5  dpi.** **3-2(A)** Sciatic nerve crush was performed on P21-P30 wild-type (N = 4) and *Etv5*-cKO (N = 3) mice on day 0 and nerves were collected on day 5. At 5  dpi, sciatic nerve sections distal to the injury were immunostained with SOX10/JUN. Blue is DAPI counterstain. Scale bars, 100 μm. **3-2(B)** The percentage of JUN^+^/DAPI^+^ Schwann cells was quantified. P-values calculated with an unpaired t-test. ns = non-significant. Data from male and female mice were mixed in this analysis. Download Figure 3-2, TIF file.

Since *Etv5*-KO mice display a progressive decline in germ cell layers and sperm production with age (Zhang et al., 2021), we asked whether *Etv5*-cKO Schwann cells had the same progressive deterioration. A sciatic nerve crush was generated in 6-month-old wild-type and *Etv5*-cKO mice, for Schwann cell quantification in the uninjured control nerve and at 5 dpi at the site distal to injury. At 5 dpi, Schwann cell numbers increased in 6-month-old wild-type male nerves (Fig. 3*F*,*G*) and trended higher in female nerves (Extended Data Fig. 3-1*D,E*), although this response was attenuated compared with P21–P30. Strikingly, Schwann cell numbers were not significantly elevated in male or female *Etv5*-cKOs at 5 dpi, even though an equivalent proportion of Schwann cells were proliferating (Fig. 3*F*,*G*; Extended Data Fig. 3-1*D,E*). These data suggest that *Etv5* is required for Schwann cell expansion post-injury, especially in mature male mice.

### Normal Schwann cell repair responses in *Etv5*-cKO mice after sciatic nerve crush

To determine whether Schwann cells acquire a repair phenotype in *Etv5*-cKO mice, we first examined the expression of JUN in P21–P30 animals at 5 d after a sciatic nerve crush. JUN is a key TF expressed in repair Schwann cells after nerve injury that plays a crucial role in Schwann cell reprogramming, promoting myelin clearance, axonal regeneration, and neuronal survival (Jessen and Mirsky, 2021). We found that JUN was expressed in a similar number of SOX10^+^ Schwann cells in wild-type and *Etv5*-cKO nerves at 5 dpi (Extended Data Fig. 3-2*A,B*).

**Figure 4.**
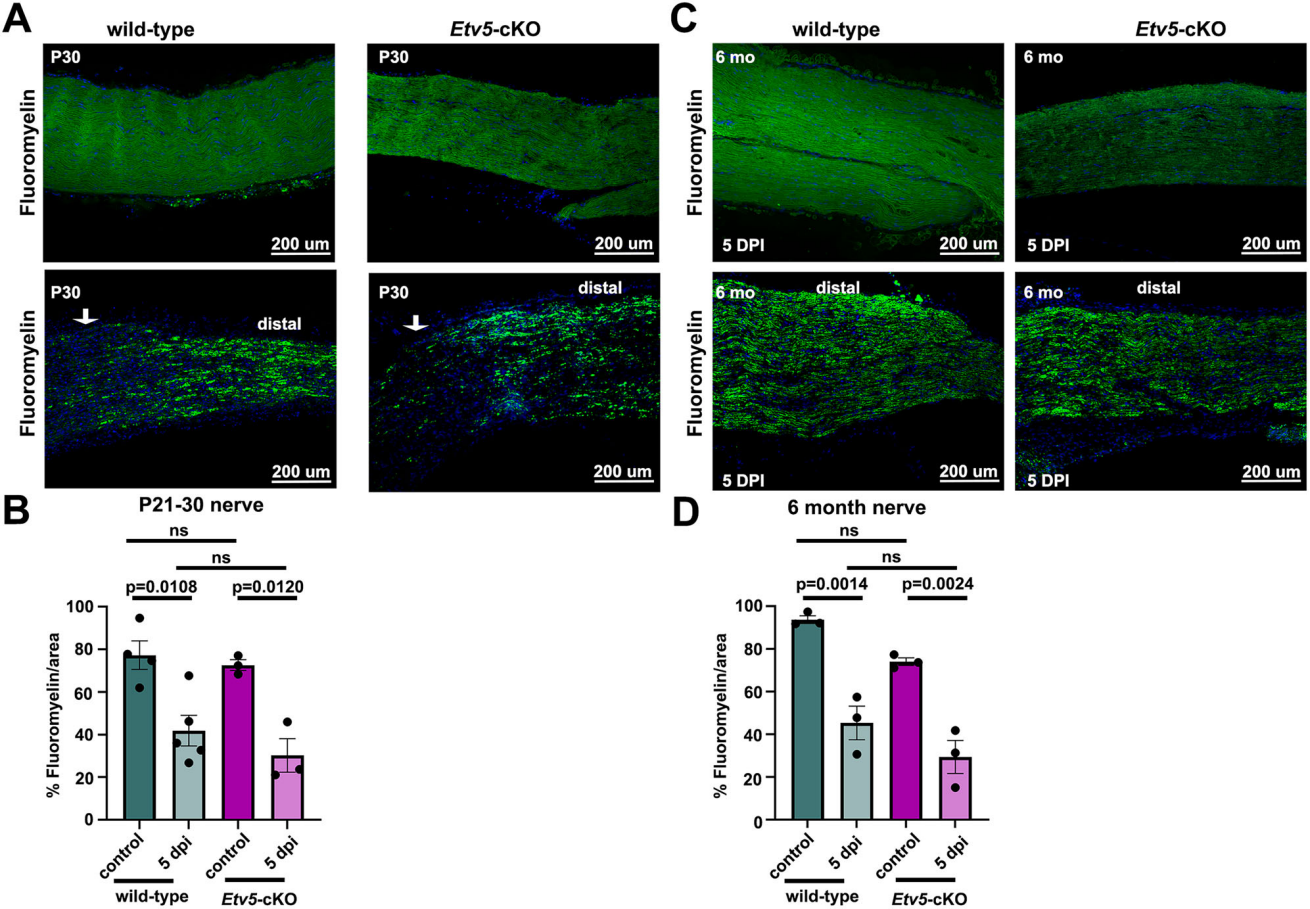
Normal myelin clearing response of male *Etv5*-cKO mice after sciatic nerve crush. ***A***, FluoroMyelin labeling of male P21–30 wild-type and *Etv5*-cKO uninjured nerves and at 5 dpi. Arrows mark the crush site, with counts performed distal to the injury site. Scale bars, 200 μm. ***B***, Quantification of FluoroMyelin density/area in P 21–30 wild-type and *Etv5*-cKO uninjured nerves and at 5 dpi, collected from male mice (*N* = 5 for wild-type and *N* = 3 for *Etv5*-cKO). *p* values calculated with one-way ANOVA and post hoc Tukey’s test. ns, nonsignificant. ***C***, FluoroMyelin labeling of 6-month-old wild-type and *Etv5*-cKO uninjured nerves and at 5 dpi collected from male mice. Counts were performed distal to the injury site. Scale bars, 200 μm. ***D***, Quantification of FluoroMyelin density/area in 6-month-old wild-type and *Etv5*-cKO uninjured nerves and at 5 dpi, collected from male mice (*N* = 3/genotype). *p* values calculated with one-way ANOVA and post hoc Tukey’s test. ns, nonsignificant.

We next examined whether myelin clearance occurs normally in *Etv5*-cKO mice. Myelin is normally cleared after sciatic nerve injury through Wallerian degeneration, a process essential for nerve regeneration, and which is conducted by repair Schwann cells and immune cells, particularly macrophages and neutrophils. To examine myelin clearance, we labeled the myelin sheath with FluoroMyelin Green, a myelin-selective, water-soluble fluorescent dye. In male wild-type and *Etv5*-cKO mice at P30 (juvenile; Fig. 4*A*,*B*) and 6 months of age (mature adult; Fig. 4*C*,*D*), FluoroMyelin Green labeled the length of the dissected sciatic nerve, with a similar density. After a sciatic nerve crush, FluoroMyelin Green labeling density declined at P30 (Fig. 4*A*,*B*) and 6 months of age (Fig. 4*C*,*D*) in animals of both genotypes. As reduced FluoroMyelin Green staining is indicative of myelin loss, we can conclude that myelin clearance occurs normally in *Etv5*-cKO mice. Thus, even though fewer Schwann cells are present in the peripheral nerve of mature, adult, male *Etv5*-cKO mice at 5 dpi, the remaining Schwann cells are sufficient to clear myelin.

### Reduced axonal size in *Etv5*-cKO sciatic nerves without overt effects on myelination

**Figure 5.**
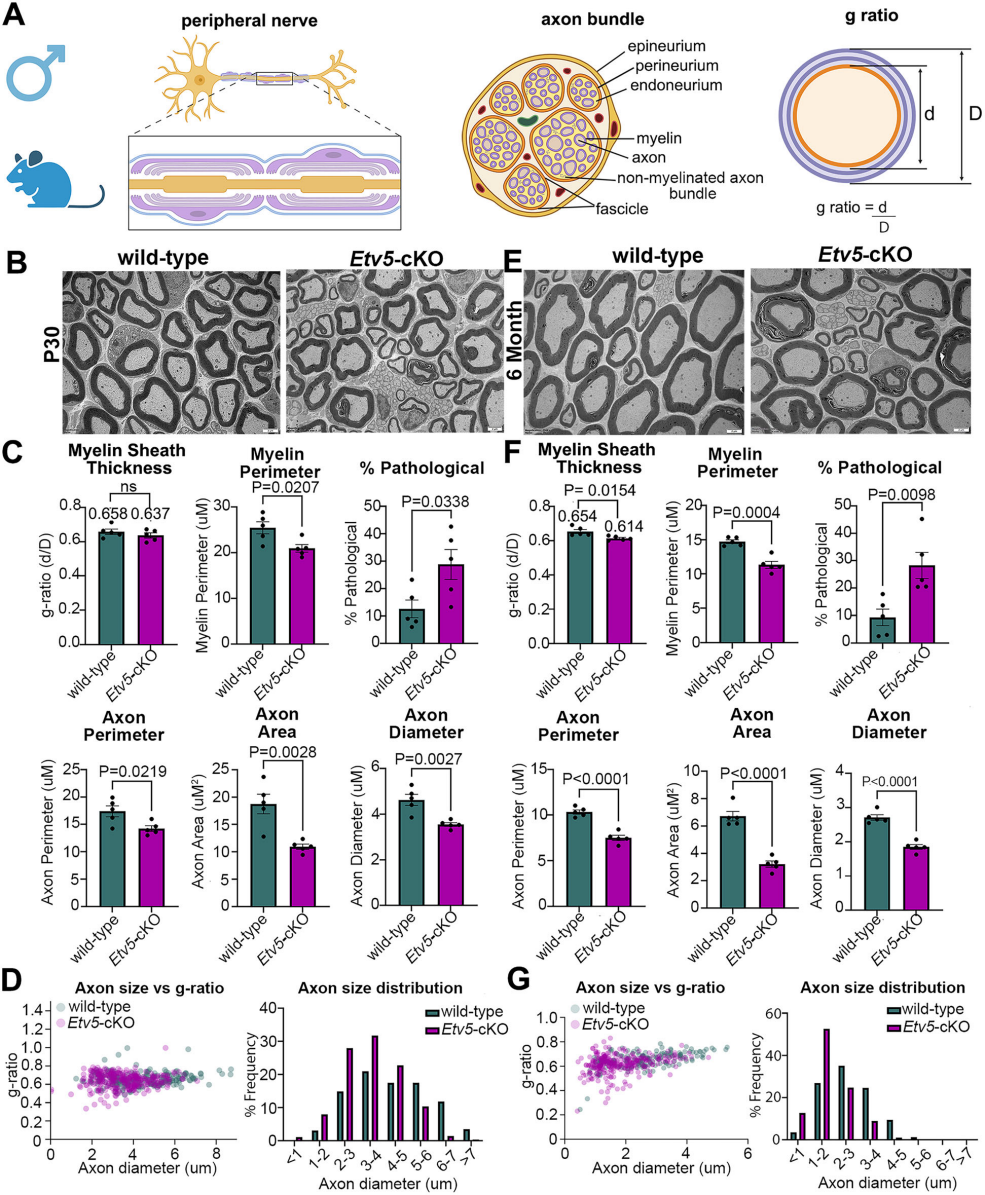
Aberrant myelination and reduced axon size in male *Etv5*-cKO sciatic nerves worsen with aging. ***A***, Experiments were conducted in male mice. Schematic illustration of peripheral nerve architecture in longitudinal and transverse planes and a depiction of the g-ratio calculation (BioRender.com). ***B–D***, TEM images of P30 sciatic nerve from wild-type and *Etv5*-cKO male mice. Scale bars, 2 μm (***B***). Quantification of the g-ratio (myelin sheath thickness), myelin perimeter, % pathological features, in addition to axon perimeter, area, and diameter. *N* = 5 nerves per genotype. *p* values calculated with an unpaired *t* test. ns, nonsignificant (***C***). Axon sizes were compared with the g-ratio and the axon size distribution was graphed for individual axons measured, totaling 229 for wild-type and 290 for *Etv5*-cKO (***D***). ***E–G***, TEM images of 6-month-old sciatic nerve from wild-type and *Etv5*-cKO male mice. Scale bars, 2 μm (***E***). Quantification of the g-ratio (myelin sheath thickness), myelin perimeter, % pathological features, in addition to axon perimeter, area, and diameter. *N* = 5 nerves per genotype. *p* values calculated with an unpaired *t* test. ns, nonsignificant (***F***). Axon sizes were compared with the g-ratio and the axon size distribution was graphed for individual axons measured, totaling 171 for wild-type and 291 for *Etv5*-cKO (***G***). Extended Data Figures 5-1 and 5-2 support this figure.

**Figure 6.**
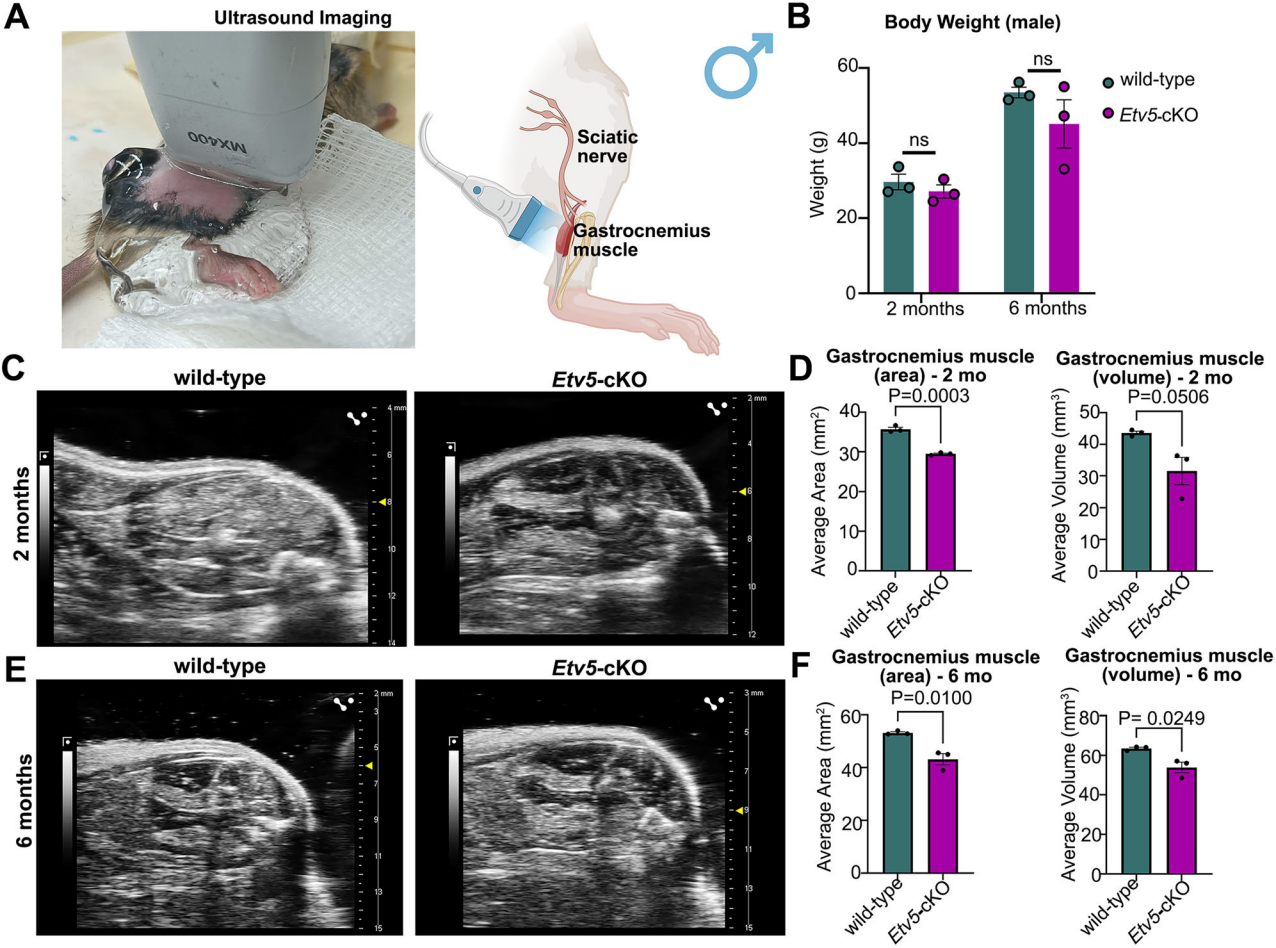
Reduced size of the gastrocnemius muscle in male *Etv5*-cKO mice. ***A***, Photo of live animal preparation for ultrasound imaging and schematic illustration of the position of the gastrocnemius muscle that was imaged in male mice (BioRender.com). ***B***, Body weight of wild-type and *Etv5*-cKO male mice at 2 and 6 months of age. *N* = 3/genotype. *p* values calculated with an unpaired *t* test. ns, nonsignificant. ***C***, ***D***, Ultrasound images of the gastrocnemius muscle from wild-type and *Etv5*-cKO male mice at 2 months of age (***C***). Quantification of the area and volume of the gastrocnemius muscle in wild-type and *Etv5*-cKO male mice at 2 months of age. *N* = 3/genotype. *p* values calculated with an unpaired *t* test. ns, nonsignificant (***D***). ***E***, ***F***, Ultrasound images of the gastrocnemius muscle from wild-type and *Etv5*-cKO male mice at 6 months of age (***E***). Quantification of the area and volume of the gastrocnemius muscle in wild-type and *Etv5*-cKO male mice at 6 months of age. *N* = 3/genotype. *p* values calculated with an unpaired *t* test. ns, nonsignificant (***F***). Extended Data Figure 6-1 supports this figure.

The peripheral nerve is composed of axon bundles that include large-diameter axons surrounded by myelin and smaller-diameter axons that remain unmyelinated (Fig. 5*A*). To visualize the detailed ultrastructure of the naive sciatic nerve, we performed transmission electron microscopy (TEM) of nerve cross sections. The compact architecture of the myelin sheath appeared similar in wild-type and *Etv5*-cKO nerves at P30 and 6 months of age, both in male and female mice. To provide a quantitative measure of myelination, and therefore nerve integrity, we used TEM images to calculate the g-ratio, which is the ratio of axon diameter (*d*) to the total myelinated nerve fiber diameter (*D*; Fig. 5*A*). We found that g-ratios were similar in wild-type (0.658 in male, 0.647 in female) and *Etv5*-cKO (0.637 in male, 0.633 in female) nerves, falling within the typical range of 0.6–0.7 (Fig. 5*B–D*; Extended Data Fig. 5-1*A,B*). By 6 months of age, the g-ratio declined in male *Etv5*-cKO mice (Fig. 5*E–G*), which also trended lower but did not reach significance in female *Etv5*-cKO mice (Extended Data Fig. 5-1*C,D*). The average myelin perimeter also declined in 6-month-old male *Etv5*-cKO mice (Fig. 5*E*,*F*). Since the g-ratio is an inverse ratio, a reduction signifies an increase in myelin thickness relative to the axon. Accordingly, we found that axon area, diameter, and perimeter were all reduced in *Etv5*-cKO male mice at P30 (Fig. 5*B–D*) and 6 months of age (Fig. 5*E–G*). Axon area and diameter also declined in female mice, but only at 6 months of age (Extended Data Fig. 5-1*A–D*). Finally, we assessed nerve fiber pathology by examining images for mitochondrial swelling, degenerated axons, debris in the axon membrane, and myelin aberrations (Dang et al., 2016; Li et al., 2018; Extended Data Fig. 5-2), focusing on male mice. Pathological features were more abundant in the nerve fiber of *Etv5*-cKO mice at P30 (Fig. 5*B*,*C*) and 6 months of age (Fig. 5*E*,*F*). Thus, *Etv5* is required to preserve axonal integrity, with more severe deficits observed in male *Etv5*-cKO mice at older ages.

10.1523/ENEURO.0410-20.2025.f5-1 Figure 5-1 **Aberrant myelination and reduced axon size in female *Etv5-* cKO sciatic nerves worsen with aging.** **5-1(A-B)** Schematic showing experiments were performed in female mice (BioRender.com). TEM images of P30 sciatic nerve from wild-type and *Etv5-*cKO female mice. Scale bars, 2 μm (A). Quantification of the g-ratio (myelin sheath thickness), axon area and axon diameter. N = 3 per genotype. P-values calculated with an unpaired t-test. ns = non-significant (B). **5-1(C-D)** TEM images of 6-month-old sciatic nerve from wild-type and *Etv5-*cKO female mice. Scale bars, 2 μm (C). Quantification of the g-ratio (myelin sheath thickness), axon area and axon diameter. N = 2 for wild-type and N = 3 for *Etv5*-cKO. P-values calculated with an unpaired t-test. ns = non-significant (D). Download Figure 5-1, TIF file.

10.1523/ENEURO.0410-20.2025.f5-2 Figure 5-2 **Pathological criteria used to assess pathological profiles in TEM images.** **5-2 Photo shows examples of pathological profiles quantified in TEM images. These include** myelin abnormalities, axoplasmic accumulation of debris, axon degeneration and mitochondrial swelling. Download Figure 5-2, TIF file.

### Reduced size of the gastrocnemius muscle in 6-month-old *Etv5*-cKO mice

Abnormalities and dysfunction of the sciatic nerve can lead to muscle denervation in addition to subsequent muscle atrophy in the lower leg and foot. We investigated whether the observed changes in the sciatic nerve structure in *Etv5*-cKO mice influenced the gastrocnemius muscle, which is innervated by the tibial nerve branch of the sciatic nerve. Ultrasound imaging was used to measure the cross-sectional area (CSA) and volume of the gastrocnemius muscle in animals that were serially imaged at 2 and 6 months of age (Fig. 6*A*; Extended Data Fig. 6-1*A*). While body weight can influence muscle mass, no differences were observed in the average weights of wild-type and *Etv5*-cKO male and female mice weighed at 2 and 6 months of age (Fig. 6*B*; Extended Data Fig. 6-1*B*). For consistency, the largest CSA of the muscle was used as the reference center midpoint to measure the area and volume of the largest part of the muscle. In male *Etv5*-cKO mice analyzed at 2 months (Fig. 6*C*,*D*) and 6 months of age (Fig. 6*E*,*F*), gastrocnemius muscle area and volume were reduced compared with control mice. In contrast, in female *Etv5*-cKO mice, both the area and volume of the gastrocnemius muscle were similar to control mice at 2 months of age and only began to decline by 6 months of age in *Etv5*-cKO mice (Extended Data Fig. 6-1*C–F*). Thus, consistent with the delayed appearance of sciatic nerve abnormalities in female *Etv5*-cKO mice, atrophy of the gastrocnemius muscle occurred later compared with male mice.

10.1523/ENEURO.0410-20.2025.f6-1 Figure 6-1 **Reduced size of the gastrocnemius muscle in *Etv5*-cKO female mice at 6-months-of-age.** **6-1(A)** Photo of live animal preparation for ultrasound imaging, and schematic illustration of the position of the gastrocnemius muscle that was imaged in female mice (BioRender.com). **6-1(B)** Body weight of wild-type and *Etv5*-cKO female mice at 2-months and 6-months-of- age. N = 3/genotype. P-values calculated with an unpaired t-test. ns = non-significant. **6-1(C,D)** Ultrasound images of the gastrocnemius muscle from wild-type and *Etv5-*cKO female mice at 2-months-of-age (C). Quantification of the area and volume of the gastrocnemius muscle in wild-type and *Etv5-*cKO female mice at 2-months-of-age. N = 3/genotype. P-values calculated with an unpaired t-test. ns = non-significant (D). **6-1(E,F)** Ultrasound images of the gastrocnemius muscle from wild-type and *Etv5-*cKO female mice at 6-months-of-age (E). Quantification of the area and volume of the gastrocnemius muscle in wild-type and *Etv5-*cKO female mice at 6-months-of-age. N = 3/genotype. P-values calculated with an unpaired t-test. ns = non-significant (F). Download Figure 6-1, TIF file.

### No defects in motor behavior in *Etv5*-cKO mice

**Figure 7.**
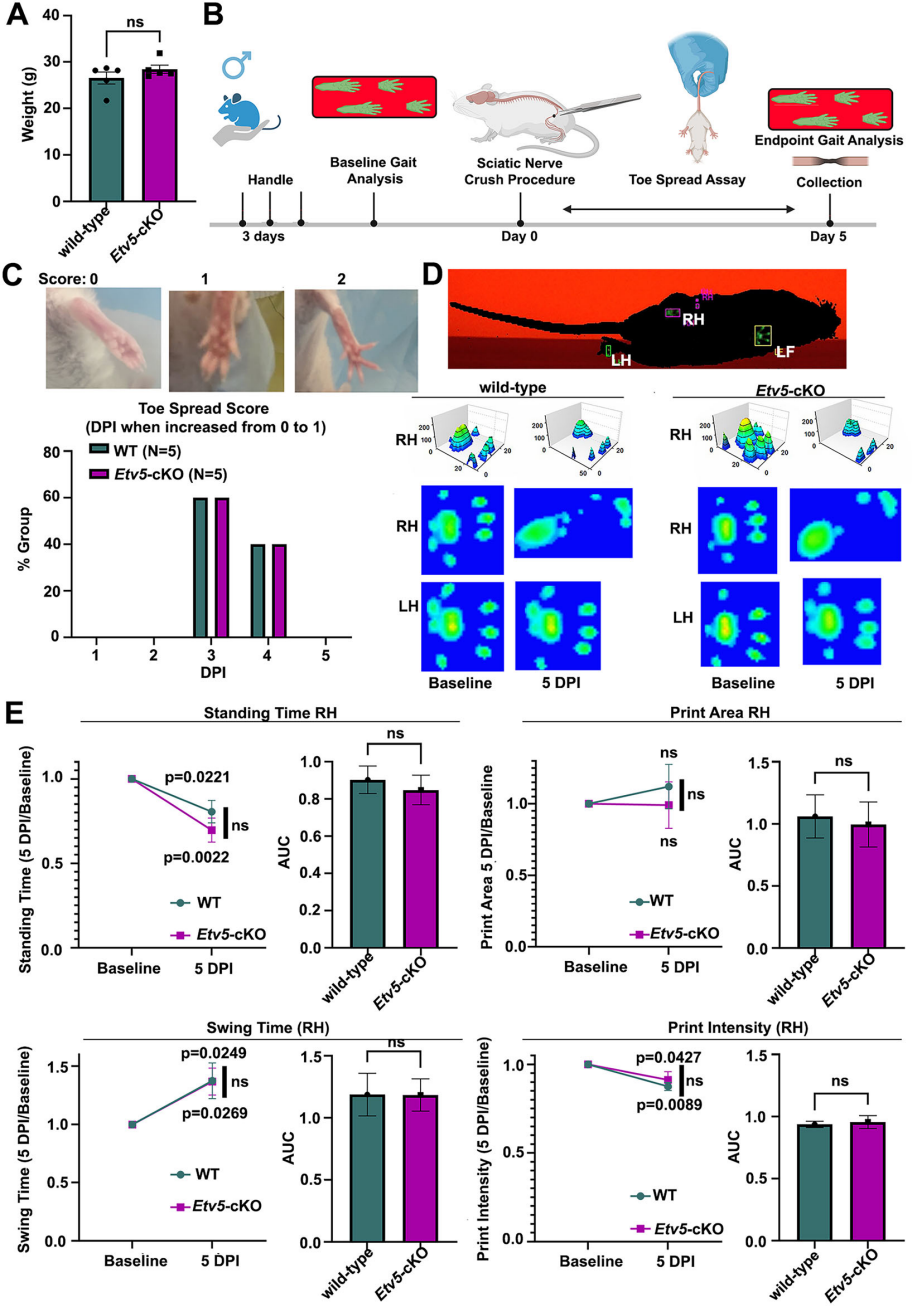
Motor deficits are similar after peripheral nerve injury in wild-type and *Etv5*-cKO male mice. ***A***, Body weight of wild-type and *Etv5*-cKO male mice used for behavioral testing at 2–2.5 months of age. *N* = 5/genotype. *p* values calculated with an unpaired *t* test. ns, nonsignificant. ***B***, Timeline of steps in the behavioral assessment (BioRender.com). Male mice were used. ***C***, Sample images of the hindlimb, with representative images for toe spread scores of 0, 1, and 2. Graph represents % of each cohort (male wild-type or male *Etv5*-cKO at 2–2.5 months of age) that progressed from a score of 0–1 at each dpi (*N* = 5/genotype). ***D***, Image of a mouse on the catwalk, and the collected images of the right hindlimb (RH) and left hindlimb (LH) collected at baseline and 5 dpi in 2–2.5-month-old wild-type and *Etv5*-cKO male mice (*N* = 5/genotype). ***E***, Calculation of the standing time, print area, swing time, and print intensity of the RH in 2–2.5-month-old wild-type and *Etv5*-cKO male mice at baseline and 5 dpi, normalized to baseline values. *p* values were calculated with a repeated-measures ANOVA. Area under the curve (AUC) measurements were made to compare the two time points. *N* = 5/genotype, *p* values calculated with an unpaired *t* test. ns, nonsignificant. Extended Data Figure 7-1 supports this figure.

*Etv5*-cKO mice did not display overt deficits in motor function, as they moved normally within the cage. We therefore investigated the role of *Etv5* in the response to a sciatic nerve crush injury. Normally, motor activity declines after a sciatic nerve crush due to demyelination and axonal degeneration. To assess the Schwann cell injury response in *Etv5*-cKO mice, we performed motor behavior tests using cohorts of 10 wild-type and 10 *Etv5*-cKO mice at 2–2.5 months of age, divided into male and female groups of five animals each. No differences were observed in the average weight of the male or female wild-type compared with *Etv5*-cKO animals used in this study (Fig. 7*A*; Extended Data Fig. 7-1*A*). Mice were first handled for 3 consecutive days before performing baseline gait analysis (Fig. 7*B*; Extended Data Fig. 7-1*B*). After 1–2 d, a sciatic nerve crush injury was performed. To assess the impact of the crush injury, we first performed a toe spread analysis on consecutive days between Day 1 and Day 5 post-injury. In uninjured mice, a suspended mouse has a reflex which fully extends all toes (assigned a score of 2), while immediately post-sciatic nerve injury, there is no toe movement when suspended (assigned a score of 0), with improvement of partially extended toes (assigned a score of 1) as an intermediate step in the recovery response (Fig. 7*C*). We monitored when the toe spread score increased from 0 to 1, showing a peak at 3 dpi in male and female wild-type and *Etv5*-cKO mice, with slightly more variability in recovery in *Etv5*-cKO female mice (Fig. 7*C*; Extended Data Fig. 7-1*C*). Nevertheless, the toe spread score increased similarly regardless of genotype or sex, suggesting that the Schwann cell repair response has initiated.

10.1523/ENEURO.0410-20.2025.f7-1 Figure 7-1 **Motor deficits are similar after peripheral nerve injury in wild-type and *Etv5*-cKO female mice.** **7-1(A)** Body weight of wild-type and *Etv5*-cKO female mice used for behavioral testing at 2- 2.5 months-of-age. N = 5/genotype. P-values calculated with an unpaired t-test. ns = non- significant. **7-1(B)** Timeline of steps in the behavioural assessment (BioRender.com). Female mice were used. **7-1(C)** Graph represents % of each cohort (female wild-type or female *Etv5-*cKO at 2-2.5 months-of-age) that progressed from a score of 0 to 1 at each dpi (N = 5/genotype). **7-1(D)** Calculation of the standing time, print area, swing time and print intensity of the RH in 2-2.5-month-old wild-type and *Etv5-*cKO female mice at baseline and 5  dpi, normalised to baseline values. P-values were calculated with a repeated measures ANOVA. Area under the curve (AUC) measurements were made to compare the two time points. N = 5/genotype, p- values calculated with an unpaired t-test. ns = non-significant. Download Figure 7-1, TIF file.

Next, we used the Catwalk gait analysis system to analyze various gait parameters, including standing time, print area, swing time, and print intensity. We focused on the right hindlimb (RH), which was subjected to the nerve crush injury, and compared baseline and 5 dpi measurements (Fig. 7*D*). Values were normalized to baseline to visualize changes in the parameters measured. The normal injury response was associated with a decrease in stand time and print intensity, as well as an increase in swing time for both male and female animals of both genotypes (Fig. 7*E*; Extended Data Fig. 7-1*D*). Though the footprint of the injured paw changed morphology after injury (Fig. 7*D*), the calculated area did not change significantly from baseline (Fig. 7*E*; Extended Data Fig. 7-1*D*). Motor deficit changes from baseline at 5 dpi were similar in *Etv5*-cKO and wild-type animals (Fig. 7*E*; Extended Data Fig. 7-1*D*), though female *Etv5*-cKO mice had a significantly reduced stand time compared with wild-type littermates (Extended Data Fig. 7-1*D*). Notably, there were no significant differences in motor deficits of *Etv5*-cKO versus wild-type mice based on area-under-the-curve (AUC) measurements (Fig. 7*E*; Extended Data Fig. 7-1*D*) in both sexes. Thus, the toe spread reflex and gait parameters measured were relatively similar in 2–2.5-month-old wild-type and *Etv5*-cKO mice at 5 dpi. Taken together, these data suggest that there are no gross differences in motor deficits in *Etv5*-cKO mice or in the initial repair response post-sciatic nerve injury, at least as assessed with these behavioral assays.

## Discussion

In this study, we investigated *Etv5* function during Schwann cell development and maturation, by first using a genetic mutant that deletes exons 2–5 (*Etv5^tm1Kmm^*; Chen et al., 2005). We were precluded from using an *Etv5* null mutant allele that removes the DNA binding domain (*Etv5^tm1Hass^*) to study Schwann cell development, as this allele results in early embryonic lethality by E8.5 (Lu et al., 2009). Nevertheless, *Etv5^tm1Kmm^* mice have a striking reduction in spermatogonial stem cell self-renewal (Chen et al., 2005), suggesting that the reduced levels of *Etv5* in this hypomorphic allele can have functional consequences. Further support for the designation of *Etv5^tm1Kmm^* as a hypomorphic allele comes from the lack of a developmental kidney defect in *Etv5^tm1Kmm^* mice (Lu et al., 2009), whereas the generation of chimeric embryos using *Etv5^tm1Hass^* embryonic stem cells revealed that *Etv5* is required for kidney development (Kuure et al., 2010). To further study *Etv5* function in postnatal stages using the equivalent of a null allele, we generated Schwann cell-specific *Etv5*-cKO mice using a *Sox10-Cre* driver.

Using a panel of well-annotated Schwann cell markers, no defects in the initial production of Schwann cells were observed in *Etv5^tm1Kmm^* peripheral ganglia analyzed at E12.5, E15.5, and E18.5, nor in the sciatic nerve of Schwann cell-specific *Etv5*-cKO mice analyzed at P21–P30. To further study *Etv5* function, we used a sciatic nerve crush model. We observed a progressive decline in the acute peripheral nerve injury response of *Etv5*-cKO mice, with fewer Schwann cells responding to injury by 6 months of age. These data suggest that *Etv5* is a positive regulator of the injury response, which is in keeping with the role for upstream ERK1/2 signaling in inducing Schwann cell myelination (Ishii et al., 2013; Sheean et al., 2014), including after injury (Harrisingh et al., 2004; Guertin et al., 2005; Napoli et al., 2012; Kim et al., 2013). Furthermore, even in the absence of injury, deficits in myelination and axonal size were observed in sciatic nerves of *Etv5*-cKO mice, correlating with atrophy of the gastrocnemius muscle, which presented earlier in male mice. However, there were similar motor deficits post-sciatic nerve crush in juvenile *Etv5*-cKO mice at 2–2.5 months of age. Although *Etv5*-cKO animals trended worse in the parameters analyzed, a possible explanation for the absence of significant behavior differences may be due to the lack of sensitivity of the measures used to detect slight changes.

MEK/ERK signaling is crucial for Schwann cell differentiation (Newbern et al., 2011) and has been implicated in the myelination process (Ishii et al., 2013), in part by inducing the expression of promyelinating TFs such as *Yy1* (He et al., 2010) as well as in promoting a dedifferentiated Schwann cell state post-injury (Napoli et al., 2012; Kim et al., 2013). The Fgf-synexpression group of Ets-domain TFs (*Etv1*, *Etv4*, *Etv5*) are also activated downstream of MEK/ERK signaling and may compensate for one another to some extent, as shown for *Etv4* and *Etv5* in kidney development (Kuure et al., 2010). While *Etv1* is expressed in myelinating Schwann cells (Srinivasan et al., 2007), there are some inconsistencies concerning its role. *Etv1* was implied not to be essential for myelinating Schwann cells and only primarily involved in non-myelinating cells (Fleming et al., 2016). In contrast, another study indicates that *Etv1* plays a positive role in myelin regulation during development and remyelination, the latter stage of repair following injury (Askar et al., 2022). Based on our data, the observed decline in the SOX10^+^ cell population in *Etv5* cKO sciatic nerves post-injury indicates that *Etv5* may serve as a positive regulator in the early stage of injury response.

We observed a reduction in axonal size and an increased incidence of structural abnormalities in naive sciatic nerves of *Etv5*-cKO mice, despite no observable reduction in Schwann cell numbers. Notably, there is a close relationship between myelinating glia and their ensheathed axons. Diphtheria toxin receptor-induced ablation of myelinating glia results in degeneration of axons prior to demyelination in both the CNS and PNS (Oluich et al., 2012; Gritsch et al., 2014). Furthermore, the deletion of genes expressed in myelinating glia, including those required for mitochondrial function, results in axonal degeneration without an overt decline in myelination (Griffiths et al., 1998; Lappe-Siefke et al., 2003; Rasband et al., 2005; Fünfschilling et al., 2012; Morrison et al., 2013; Viader et al., 2013). The initial response to axon degeneration is to make up for the decreasing axon size by thickening the myelin sheath around it (Sanders and Hardy, 1948), which could possibly explain the decrease in g-ratio observed in 6-month-old *Etv5*-cKO male mice, indicating hypermyelination. It would be interesting in future studies to see if the myelin sheath eventually becomes thinner as time progresses and axonal degeneration ensues. Proteomic protein analysis of the rodent sciatic nerve displayed sex-specific age trajectories, including in myelin-specific proteins within the naive nerve (Xian et al., 2022), and during the injury response (Barry et al., 2018). Dynamic molecular changes in tissues differ in males and females of similar ages, explaining disparities between sexes in the data presented in this study.

*Etv5* is additionally expressed in neurons in the peripheral ganglia, including TrkA^+^ sensory neurons in the DRGs, and may have neuronal-specific functions (Chotteau-Lelievre et al., 1997; Hagedorn et al., 2000; Fontanet et al., 2013). We did not assess these neuronal functions as our *Sox10-Cre* driver was glial specific. Therefore, future studies using a Cre-driver that is active in peripheral ganglia neurons, such as *Advillin-Cre* (Zurborg et al., 2011), could be performed. In summary, while our study does not support a critical role for *Etv5* in Schwann cell development, we demonstrate that *Etv5* is involved in maintaining the integrity of the nerve as well as regulating the Schwann cell repair response post-injury.

## Synthesis

Reviewing Editor: Benjamin Deneen, Baylor College of Medicine Decisions are customarily a result of the Reviewing Editor and the peer reviewers coming together and discussing their recommendations until a consensus is reached. When revisions are invited, a fact-based synthesis statement explaining their decision and outlining what is needed to prepare a revision will be listed below. The following reviewer(s) agreed to reveal their identity: Ahmet Hoke.

The reviewers have found merit and interest on the findings reported. I concur with the reviewers' comments expressing the interest of the study. However, they also expressed some concerns about several issues that need to be addressed and clarified. I also consider pertinent the concerns expressed.

Reviewers and I believe that the comments require less than two months of work.

I am looking forward to receiving a revised version of the manuscript with the reviewers´ comments addressed.

Alfonso Araque

Reviewing Editor

eNeuro

Comments of the reviewers:

Reviewer 1.

Authors in this manuscript describes for the first time a role for Etv5 in regulating the Schwann cell repair response after peripheral nerve injury. They report normal development in Etv5-/- mice but increased Schwann cell numbers at the site distal to the crush injury site. Overall, the paper is well-written with a few minor concerns.

1. Missing N numbers for all the experiments.

2. The exact site of tissues collected from distal stump is not clear. More detailed description is necessary.

3. How long does the increased Schwann cell injury responses last? Current study only had a single time point.

4. The experiments for nerve injury was carried out on P21. Would adult animals show similar responses?

5. Figure 5. The background levels for injured and uninjured images are different. Was the data analyzed based on the same background levels?

6. Were antibodies used in the study validated?

Reviewer 2.

In this study the authors examine the role of Etv5 in Schwann cell development and response to injury using a hypomorphic mutant called Etv5tm1Kmm. Overall, this small descriptive study is properly conducted, and the results are clearly explained. The data is discussed properly with attention to obvious caveats but the conclusions about knockdown of this factor could be exploited for regenerative purposes is unfounded and needs to be removed (line 315) (see below).

They show that in this Etv5 hypomorph there is no defect in development of Schwann cells, but there is an increase in Schwann cell numbers after sciatic nerve crush when denervated Schwann cells assume a "repair Schwann cell" identity. I wish they had conducted additional studies building on this finding after injury and see if this has any role in nerve regeneration. Is it possible that the increased numbers of repair Schwann cells would lead to better regenerative success? Or would you expect a detrimental effect? Do the increased numbers of Schwann cells in the distal nerve of Etv5 hypomorph mice after crush actually have repair phenotype? Is it possible that reduced Atv5 function alters the Schwann cell gene expression and make them less supportive of regeneration? Do these higher numbers of Schwann cells express c-Jun and other markers of repair cell phenotype? I think the authors at least do these simple experiments to support their conclusion that knockdown of Etv5 may have therapeutic affect (ideally, I'd like to see an actual regeneration experiment).

Minor: What is surprising is that the authors use the designation Etv5-/- for the mice even though these are not homozygous null mice (embryonically lethal). Why not just use the correct mouse designation throughout the paper and state that these are Etv5 hypomorphs?
