# Supplementary figures and images for: Etv5 Is Required for Peripheral Nerve Function and the Injury Response
Source: eNeuro. 2025 Jul 10;12(7):ENEURO.0410-20.2025. doi: 10.1523/ENEURO.0410-20.2025 (PMC12302669; doi:10.1523/ENEURO.0410-20.2025)

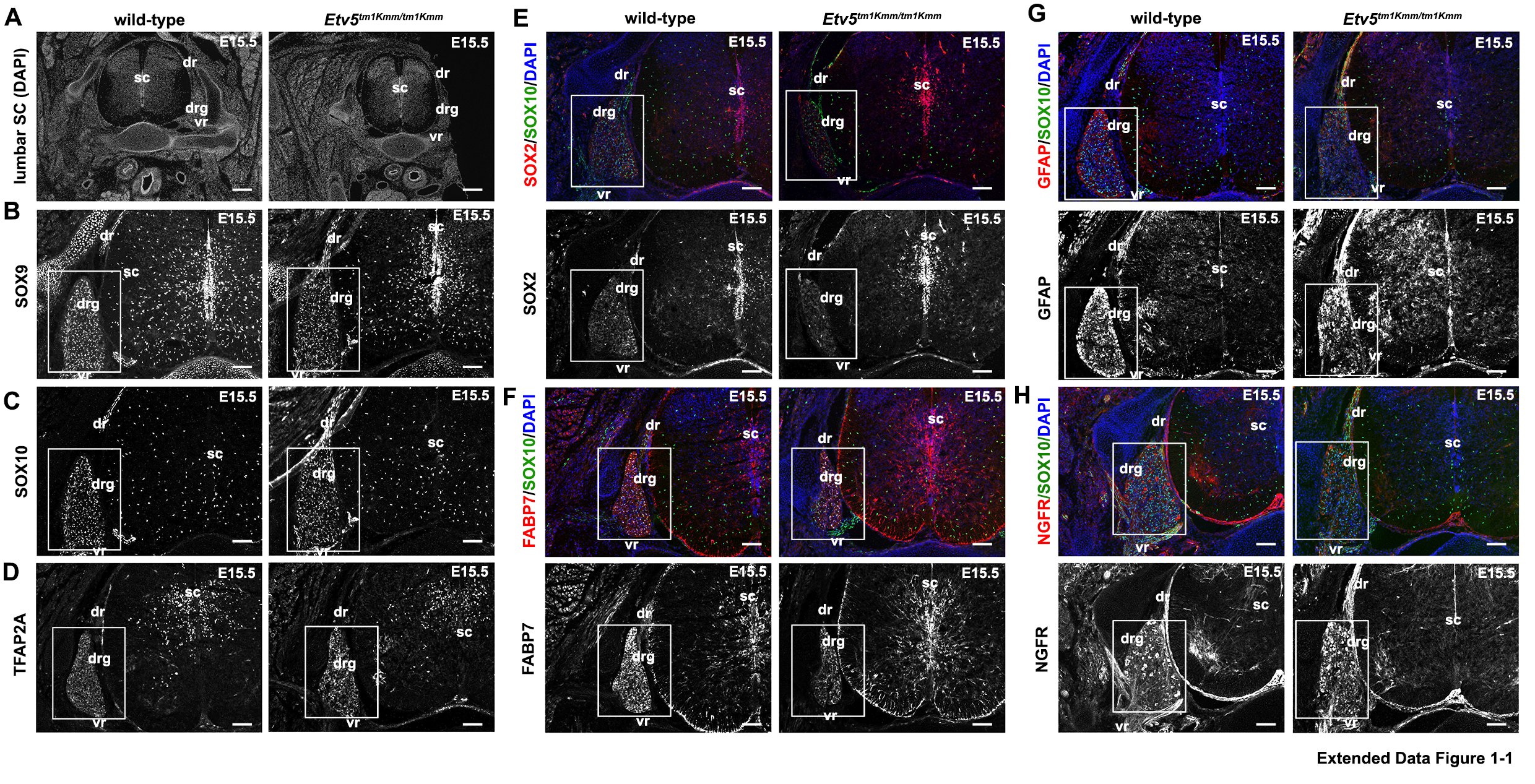

Supplement: Figure 1-1 — Schwann cell lineage markers are expressed normally in immature Schwann cells in E15.5 Etv5tm1Kmm homozygous mutant embryos. 1-1(A) Low magnification DAPI-stained images of transverse sections through the lumbar spinal cord of E15.5 wild-type and Etv5tm1Kmm homozygous embryos. Scale bars, 100 μm. 1-1(B-D) Expression of SOX9 (B), SOX10 (C) and TFAP2A (D) in transverse sections through the lumbar spinal cord of E15.5 wild-type and Etv5tm1Kmm homozygous mutant embryos. Scale bars, 60 μm. 1-1(E-H) Co-expression of SOX10 (green) with SOX2 (red, E), FABP7 (red or black/white, F), GFAP (red or black/white, G), and NGFR (red or black/white, H), counterstained with DAPI (blue) in transverse sections through the lumbar spinal cord of E15.5 wild-type and Etv5tm1Kmm homozygous mutant embryos. dr, dorsal root; drg, dorsal root ganglion; sc, spinal cord; vr, ventral root. Scale bars (A,B), 100 μm; (C-H), 60 μm. Download Figure 1-1, TIF file. [file eneuro-12-ENEURO.0410-20.2025-s002.tif]

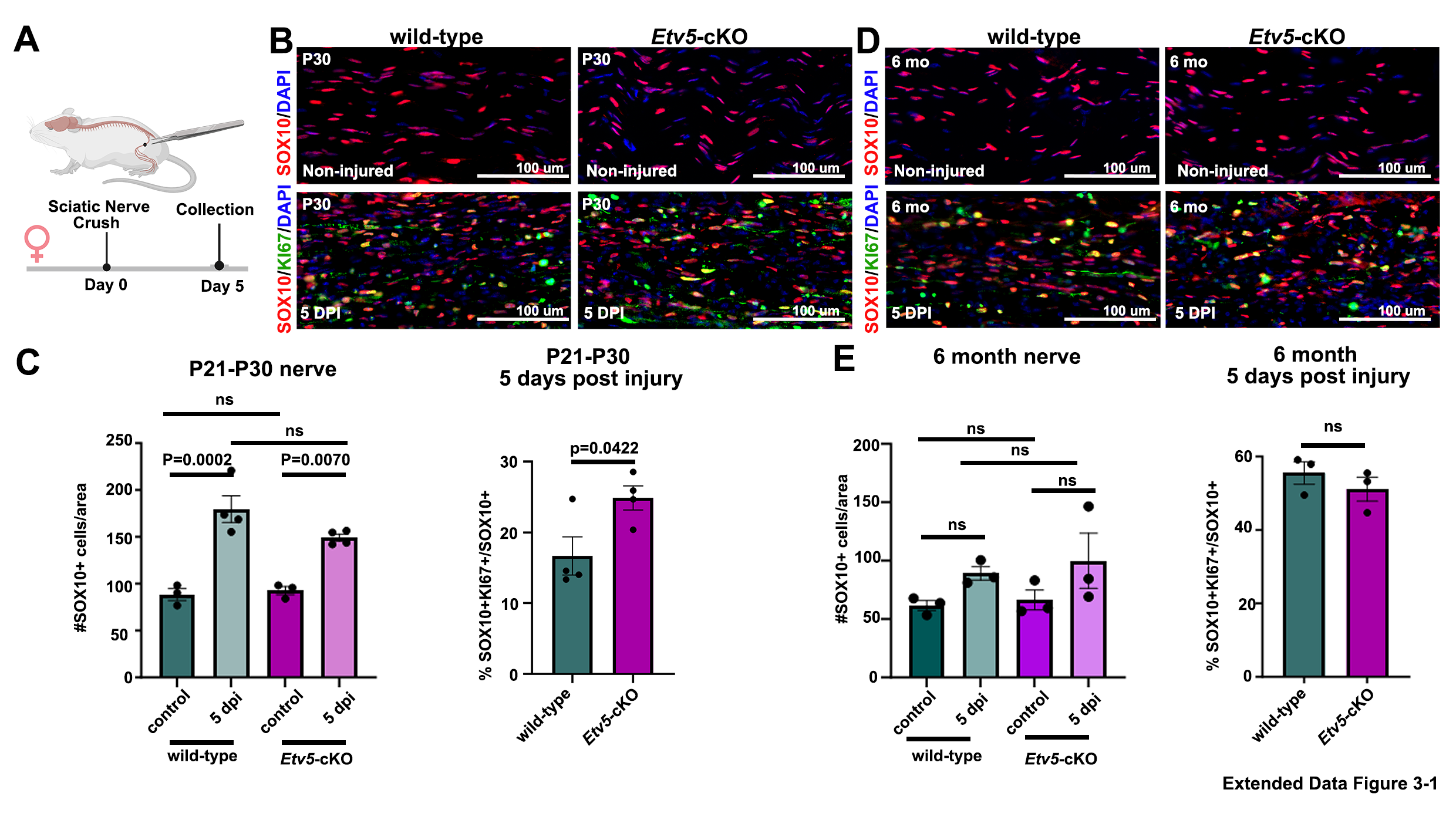

Supplement: Figure 3-1 — Defects in the response of female Etv5-cKO mice to a sciatic nerve crush. 3-1(A) Sciatic nerve crush was performed on female mice on day 0 and nerves were collected on day 5 (BioRender.com). 3-1(B-C) SOX10 (red) and KI67 (green) labeling of non-injured P 21-30 nerves and at 5-day post-injury (dpi) collected from wild-type and Etv5-cKO female mice. Blue is DAPI counterstain. Scale bars, 100 μm (B). Quantification of the percentage of SOX10+ cells/area in control, uninjured P21-30 nerves, and at 5 dpi, collected from wild-type (N = 4) and Etv5- cKO (N = 4) female mice (C). P-values calculated with one-way ANOVA and post-hoc Tukey test. Quantification of the percentage of SOX10+ cells that are proliferating (KI67+) in control, uninjured P21-30 nerves, and at 5 dpi, collected from wild-type (N = 4) and Etv5-cKO (N = 4) female mice. P-values calculated with an unpaired t-test. ns = non-significant (C). 3-1(D-E) SOX10 (red) and KI67 (green) labeling of non-injured 6-month-old nerves, and at 5-day post-injury (dpi) collected from wild-type and Etv5-cKO female mice. Blue is DAPI counterstain. Scale bars, 100 μm (D). Quantification of the percentage of SOX10+ cells/area in control, uninjured 6-month-old nerves, and at 5 dpi, collected from wild-type (N = 3) and Etv5-cKO (N = 3) female mice. P-values calculated with one-way ANOVA and post-hoc Tukey test. Quantification of the percentage of SOX10+ cells that are proliferating (KI67+) in control, uninjured 6-month-old nerves, and at 5 dpi, collected from wild-type (N = 3) and Etv5-cKO (N = 3) female mice. P-values calculated with one-way ANOVA and post-hoc Tukey test. ns = non-significant (E). Download Figure 3-1, TIF file. [file eneuro-12-ENEURO.0410-20.2025-s003.tif]

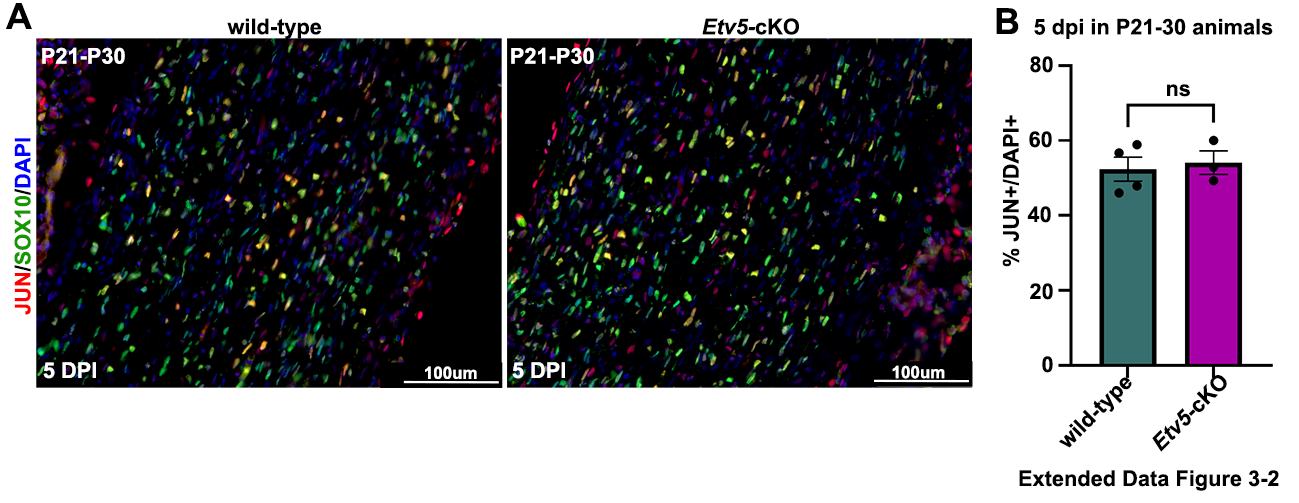

Supplement: Figure 3-2 — JUN is expressed normally in repair Schwann cells in Etv5- cKO sciatic nerves at 5 dpi. 3-2(A) Sciatic nerve crush was performed on P21-P30 wild-type (N = 4) and Etv5-cKO (N = 3) mice on day 0 and nerves were collected on day 5. At 5 dpi, sciatic nerve sections distal to the injury were immunostained with SOX10/JUN. Blue is DAPI counterstain. Scale bars, 100 μm. 3-2(B) The percentage of JUN+/DAPI+ Schwann cells was quantified. P-values calculated with an unpaired t-test. ns = non-significant. Data from male and female mice were mixed in this analysis. Download Figure 3-2, TIF file. [file eneuro-12-ENEURO.0410-20.2025-s004.tif]

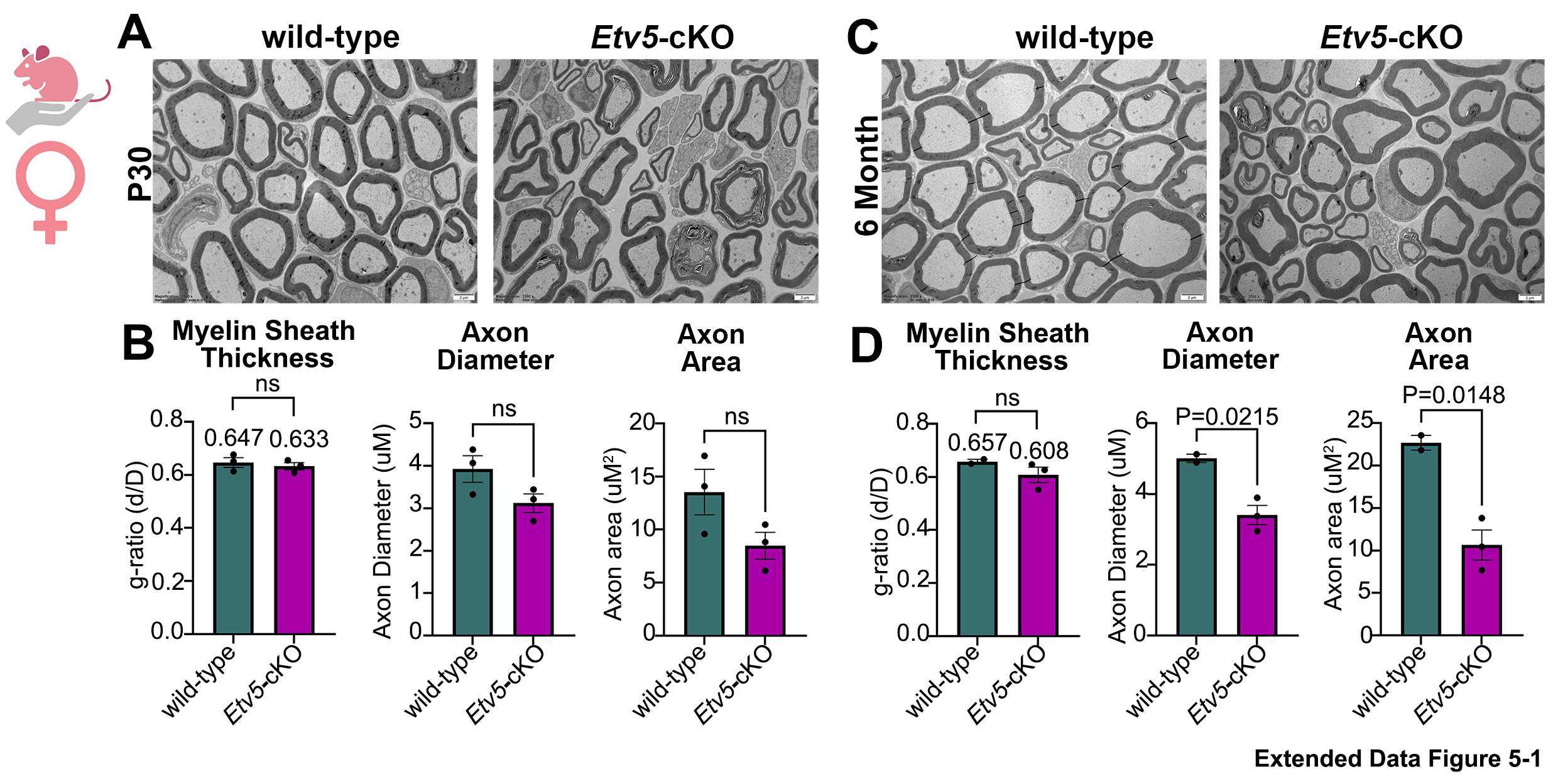

Supplement: Figure 5-1 — Aberrant myelination and reduced axon size in female Etv5- cKO sciatic nerves worsen with aging. 5-1(A-B) Schematic showing experiments were performed in female mice (BioRender.com). TEM images of P30 sciatic nerve from wild-type and Etv5-cKO female mice. Scale bars, 2 μm (A). Quantification of the g-ratio (myelin sheath thickness), axon area and axon diameter. N = 3 per genotype. P-values calculated with an unpaired t-test. ns = non-significant (B). 5-1(C-D) TEM images of 6-month-old sciatic nerve from wild-type and Etv5-cKO female mice. Scale bars, 2 μm (C). Quantification of the g-ratio (myelin sheath thickness), axon area and axon diameter. N = 2 for wild-type and N = 3 for Etv5-cKO. P-values calculated with an unpaired t-test. ns = non-significant (D). Download Figure 5-1, TIF file. [file eneuro-12-ENEURO.0410-20.2025-s005.tif]

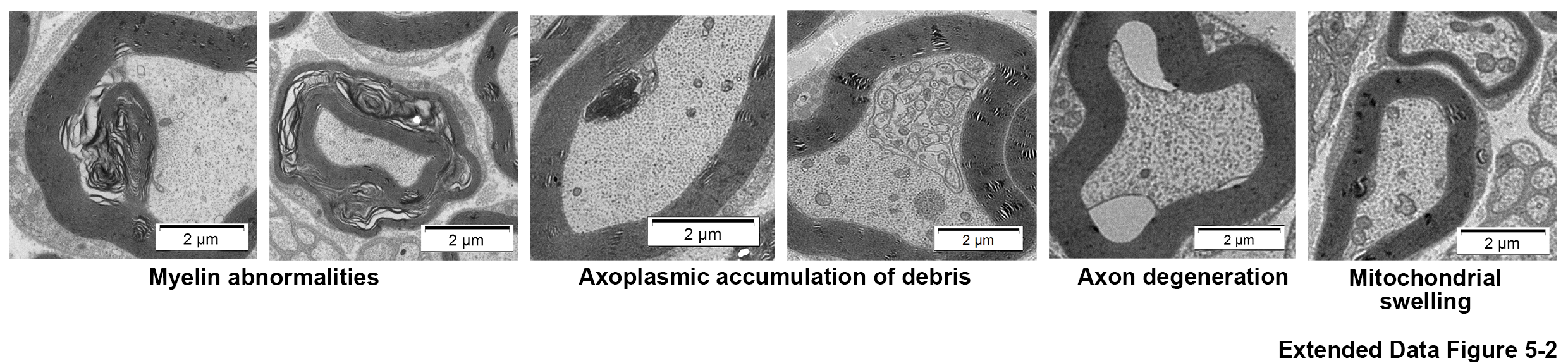

Supplement: Figure 5-2 — Pathological criteria used to assess pathological profiles in TEM images. 5-2 Photo shows examples of pathological profiles quantified in TEM images. These include myelin abnormalities, axoplasmic accumulation of debris, axon degeneration and mitochondrial swelling. Download Figure 5-2, TIF file. [file eneuro-12-ENEURO.0410-20.2025-s006.tif]

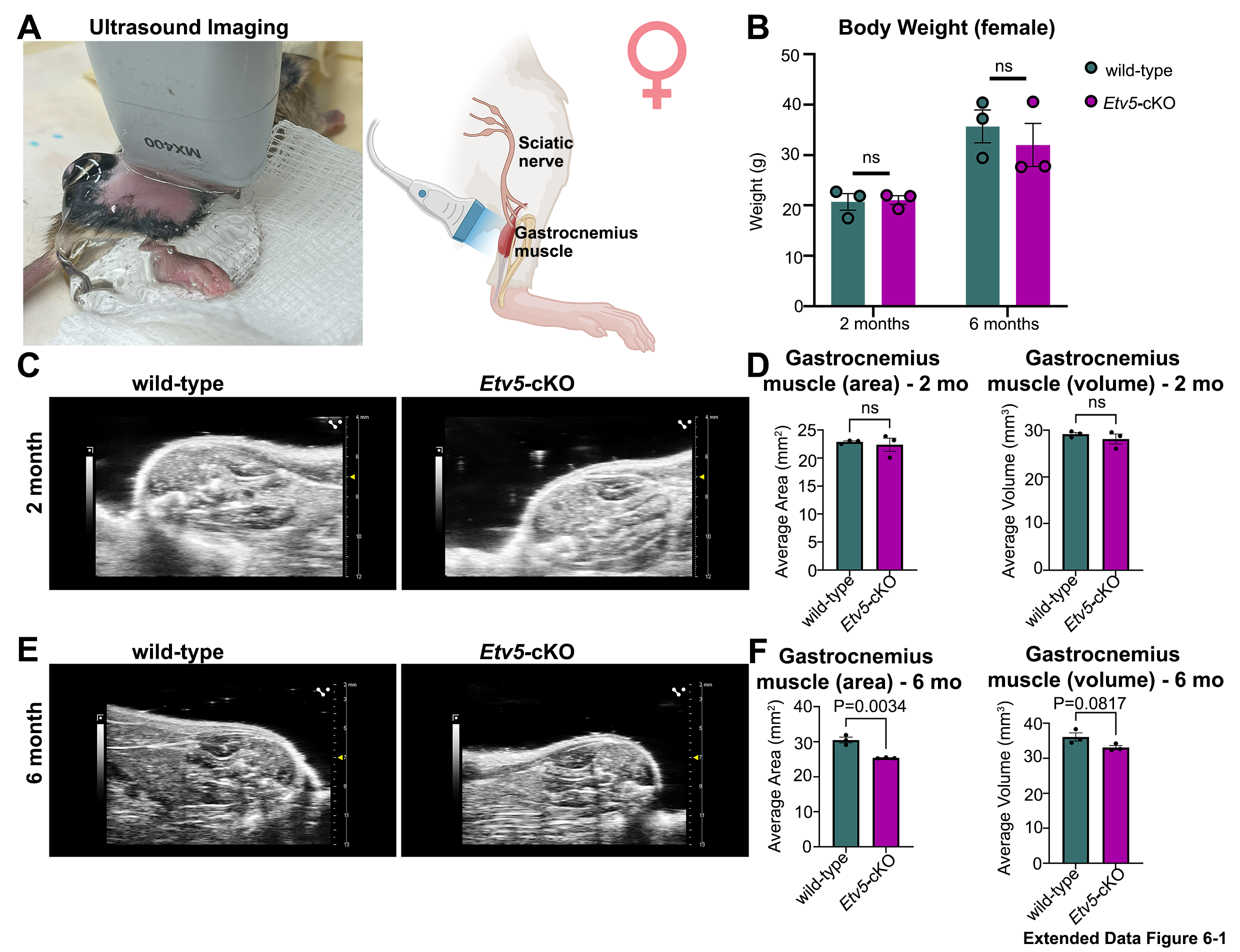

Supplement: Figure 6-1 — Reduced size of the gastrocnemius muscle in Etv5-cKO female mice at 6-months-of-age. 6-1(A) Photo of live animal preparation for ultrasound imaging, and schematic illustration of the position of the gastrocnemius muscle that was imaged in female mice (BioRender.com). 6-1(B) Body weight of wild-type and Etv5-cKO female mice at 2-months and 6-months-of- age. N = 3/genotype. P-values calculated with an unpaired t-test. ns = non-significant. 6-1(C,D) Ultrasound images of the gastrocnemius muscle from wild-type and Etv5-cKO female mice at 2-months-of-age (C). Quantification of the area and volume of the gastrocnemius muscle in wild-type and Etv5-cKO female mice at 2-months-of-age. N = 3/genotype. P-values calculated with an unpaired t-test. ns = non-significant (D). 6-1(E,F) Ultrasound images of the gastrocnemius muscle from wild-type and Etv5-cKO female mice at 6-months-of-age (E). Quantification of the area and volume of the gastrocnemius muscle in wild-type and Etv5-cKO female mice at 6-months-of-age. N = 3/genotype. P-values calculated with an unpaired t-test. ns = non-significant (F). Download Figure 6-1, TIF file. [file eneuro-12-ENEURO.0410-20.2025-s007.tif]

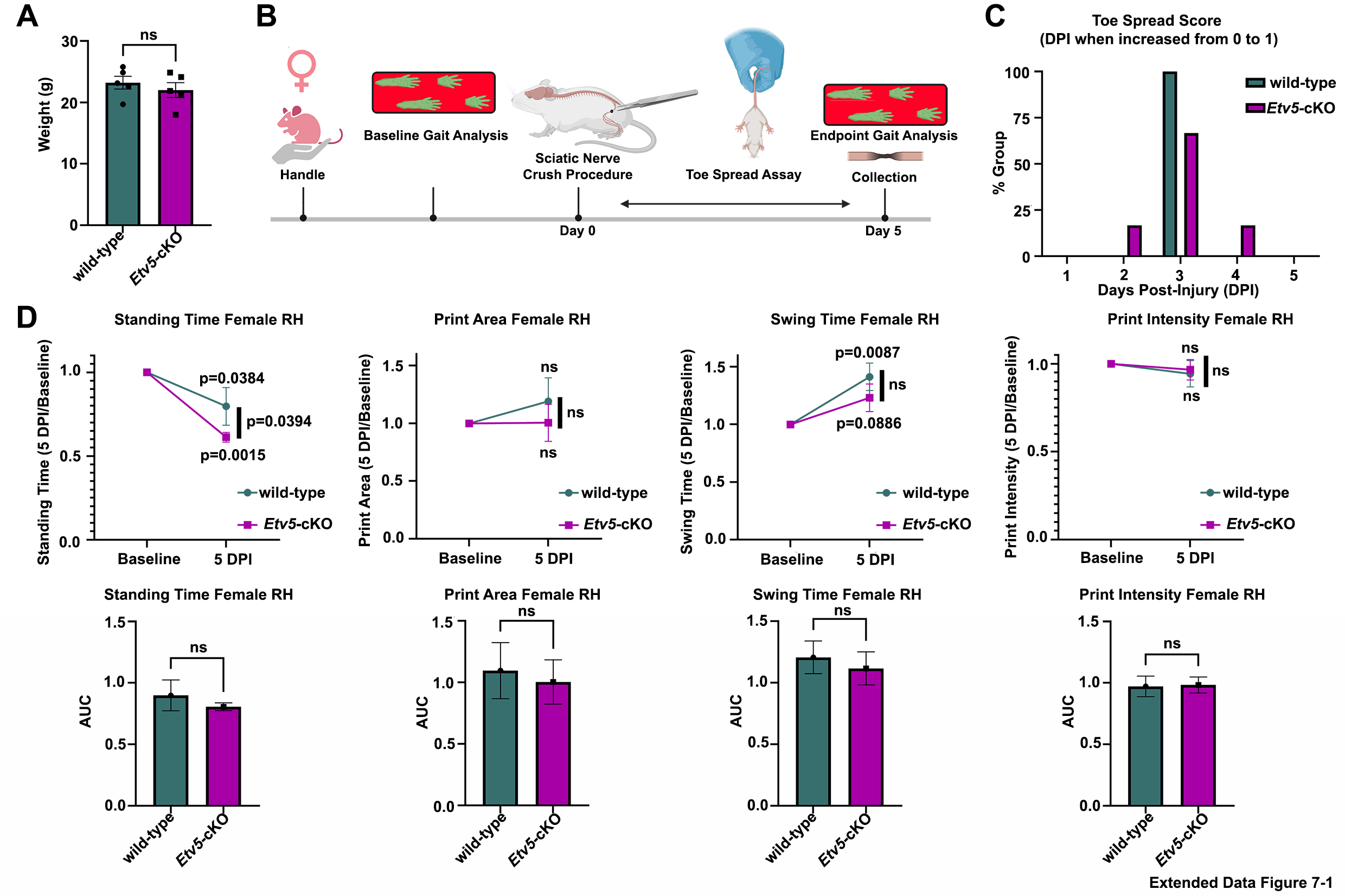

Supplement: Figure 7-1 — Motor deficits are similar after peripheral nerve injury in wild-type and Etv5-cKO female mice. 7-1(A) Body weight of wild-type and Etv5-cKO female mice used for behavioral testing at 2- 2.5 months-of-age. N = 5/genotype. P-values calculated with an unpaired t-test. ns = non- significant. 7-1(B) Timeline of steps in the behavioural assessment (BioRender.com). Female mice were used. 7-1(C) Graph represents % of each cohort (female wild-type or female Etv5-cKO at 2-2.5 months-of-age) that progressed from a score of 0 to 1 at each dpi (N = 5/genotype). 7-1(D) Calculation of the standing time, print area, swing time and print intensity of the RH in 2-2.5-month-old wild-type and Etv5-cKO female mice at baseline and 5 dpi, normalised to baseline values. P-values were calculated with a repeated measures ANOVA. Area under the curve (AUC) measurements were made to compare the two time points. N = 5/genotype, p- values calculated with an unpaired t-test. ns = non-significant. Download Figure 7-1, TIF file. [file eneuro-12-ENEURO.0410-20.2025-s008.tif]
